# Copper II Complexes Based on Benzimidazole Ligands as a Novel Photoredox Catalysis for Free Radical Polymerization Embedded Gold and Silver Nanoparticles

**DOI:** 10.3390/polym15051289

**Published:** 2023-03-03

**Authors:** Lama M. Alhomaidan, Haja Tar, Abrar S. Alnafisah, Lotfi M. Aroua, Noura KouKi, Fahad M. Alminderej, Jacques Lalevee

**Affiliations:** 1 Department of Chemistry, College of Science, Qassim University, Buraidah 51452, Saudi Arabia; 2 Laboratory of Organic Structural Chemistry and Macromolecules, Department of Chemistry, Faculty of Sciences of Tunis, Tunis El-Manar University, El Manar I, Tunis 2092, Tunisia; 3 CNRS, IS2M UMR 7361, Université de Haute-Alsace, F-68100 Mulhouse, France

**Keywords:** copper II complex, benzimidazole ligands, free radical polymerization, photoredox catalysts, gold and silver nanoparticles

## Abstract

The copper II complex’s novel benzimidazole Schiff base ligands were manufactured and gauged as a new photoredox catalyst/photoinitiator amalgamated with triethylamine (TEA) and iodonium salt (Iod) for the polymerization of ethylene glycol diacrylate while exposed to visible light by an LED Lamp at 405 nm with an intensity of 543 mW/cm^2^ at 28 °C. Gold and silver nanoparticles were obtained through the reactivity of the copper II complexes with amine/Iod salt. The size of NPs was around 1–30 nm. Lastly, the high performance of copper II complexes for photopolymerization containing nanoparticles is presented and examined. Ultimately, the photochemical mechanisms were observed using cyclic voltammetry. The preparation of the polymer nanocomposite nanoparticles in situ was photogenerated during the irradiation LED at 405 nm with an intensity of 543 mW/cm^2^ at 28 °C process. UV-Vis, FTIR, and TEM analyses were utilized for the determination of the generation of AuNPs and AgNPs which resided within the polymer matrix.

## 1. Introduction

Nanoparticle formation and characterization have been a subject of interest for a long time. This is because of the exclusive features and potential applications of these particles in various fields, including microelectronics [1], photocatalysis [2], magnetic devices [3], and powder metallurgy [4]. Metallic nanoparticles’ size, shape, structure, morphology, and crystallinity [5] are crucial determinants of their intrinsic properties. Noble metal nanoparticles show significant promise for technological and biological use, especially when incorporated into a polymer matrix.

The combination of metal nanoparticles and organic polymers creates hybrid materials with novel properties [6], such as their ability to generate enhanced electromagnetic fields and excitation of localized surface plasmon resonances (LSPR) [7] enhancements in the modulus and melt strengths, higher thermal stability, and heat resistance [8]. These properties are influenced by the size and shape of the metal nanoparticles, which can be controlled through chemical methods. One example is spherical silver nanoparticles, which strongly absorb the visible light region due to their surface plasmon resonance. This occurs because of their small size and spherical shape [9,10,11,12].

Metal nanoparticles are generated within the polymer matrix through cationic precursors, which have high dispersibility and undergo simple chemical reactions [13] or photochemical reductions [9]. Photochemical synthesis is a powerful tool for producing nanomaterials due to its precise control over space and time. This also makes it easy to apply imaging and a lithographic technique, as light is an essential component of nanostructure production [14]. The photochemical reduction approach involves irradiation with a dye sensitizer, which can also be used to make polymer-embedded nanoparticles in the presence of silver ions [15,16,17,18,19]. For example, L. Balan et al. studied the formation of silver nanoparticles in a polyacrylate polymer via cross-linking photopolymerization of pentaerythritol triacrylate monomer and using eosin dye as a visible photosensitizer [20]. The obtained nanocomposite exhibits spherical-shaped Ag nanoparticles whose homogeneous dispersion is ascribable to the capping effect of monomer acrylate branches that are not engaged in the polymer network.

Additionally, the utilization of copper nanoparticles has been demonstrated to have potential applications, as reported by S. Giuffrida et al. [21] in their investigation of copper nanoparticle integration into poly(vinyl pyrrolidone). The plasmon resonance of metal nanoparticles governs their optical response in the visible region of the spectrum. S. Mitzscherling et al. [22] reported that the dielectric function of gold nanoparticles is dissimilar to that of bulk gold. Several research groups have used Type I and Type II photoinitiators to form Ag, Au, and Cu nanoparticles photochemically, including in situ formation of NPs in the polymer matrix. Their photochemistry was analyzed [23,24,25,26,27,28,29]. Recently, a fast method has been developed to produce AuNP/polymer composite materials by reducing HAuCl4 salt and simultaneously creating a polymer matrix using UV light. Prepared AuNPs/polymer nanocoating exhibit a reflected gold mirror view depending on the irradiation time in an attempt to make them near or at the polymer surface [29]. Recently, researchers have investigated the photoinitiation ability of copper II complex-based systems for making gold nanoparticle polymer networks and for radical polymerization, as reported by H. Tar et al. [30]. In the photoinitiated system (PIS), a copper II complex and gold (III) chloride were utilized to produce radicals, cations, and gold nanoparticles within the polymer matrix.

Metal complexes, including zinc, ruthenium, iridium, and copper, have been utilized as photoinitiators for radical and cationic polymerization [31,32,33,34,35,36]. Copper is particularly appealing as a photoinitiator due to the availability of reasonably priced complexes with long-lived excited states [37,38,39,40,41,42,43,44]. In the photoinitiated system (PIS), a copper II complex and gold (III) chloride were utilized to produce radicals, cations, and gold nanoparticles within the polymer matrix.

This study explores the potential of new copper complexes based on benzimidazole Schiff base ligand as a photoredox catalyst for polymerization reactions. The study will use three-component photoinitiating systems, composed of amine, iodonium salt, and Cu (II) complexes (PIS), to produce metal nanoparticles (MNPs) photochemically. The photochemical process involved in the polymerization will be carefully analyzed using techniques such as steady-state photolysis, cyclic voltammetry, and free energy changes. Finally, the study provides a practical approach in which the EGDA photopolymerization and the simultaneous in situ reduction of silver nitrate and HAuCl4 to AgNPs and AuNPs are carried out, resulting in an EGDA polymer.

## 2. Materials and Methods

### 2.1. Materials

**Scheme 1:**
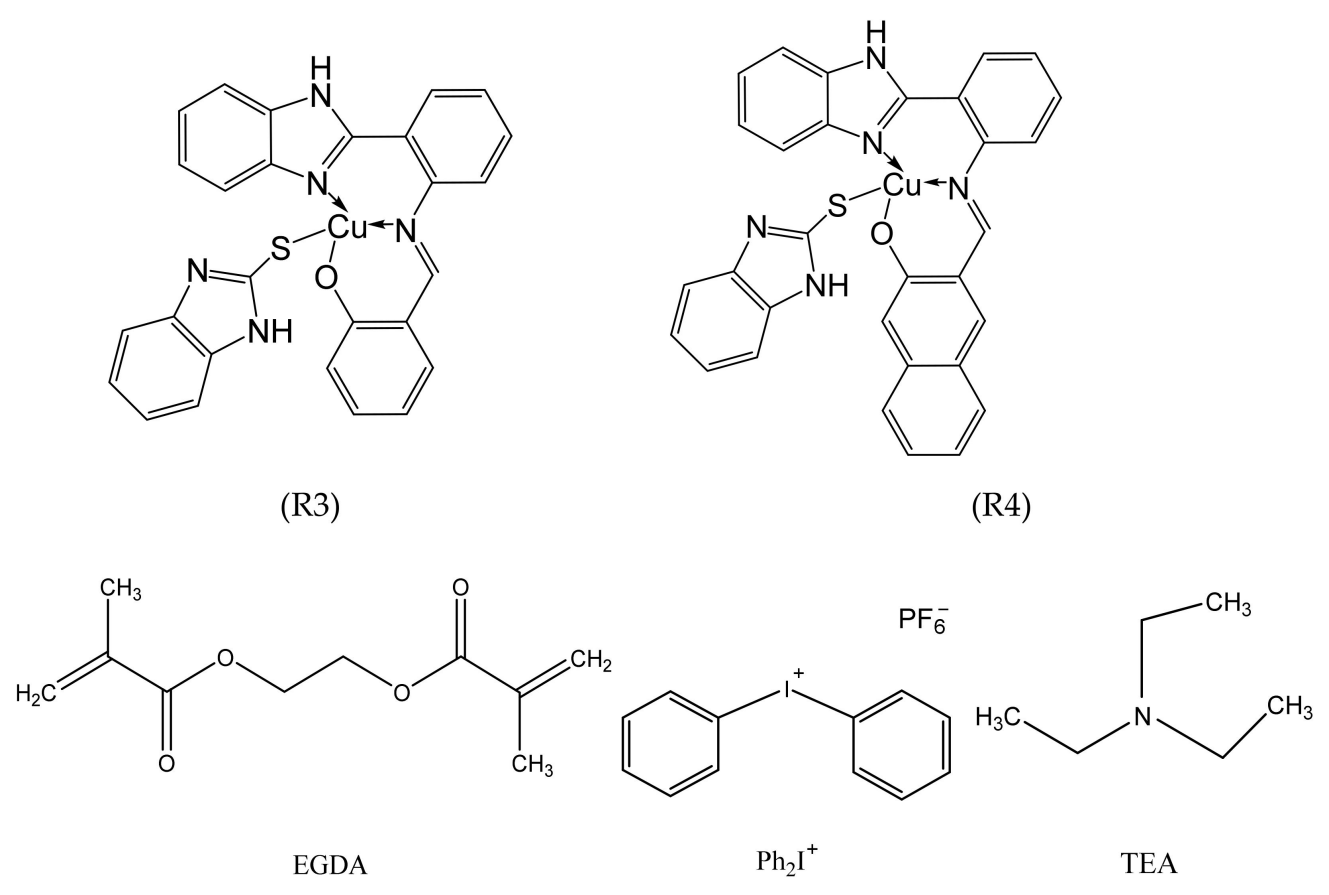
Structures of R3 and R4 copper II complexes and the monomers and additives used in this study.

The synthesis of benzimidazole ligand and its corresponding copper II complexes are represented in the supporting information. The structure of the copper II complexes (R3, R4) is presented in Scheme 1. Triethylamine (TEA), gold (III) chloride (HAuCl_4_), silver nitrate (AgNO_3_), ethylene glycol diacrylate (EGDA), and diphenyl iodonium hexafluorophosphate (Ph_2_I^+^) were purchased from Sigma Aldrich. Scheme 1 illustrates all the chemical compound structures used in the photopolymerization procedures.

### 2.2. Irradiation Source

The solution was situated in a Pyrex tube ((i.d.) 9 mm) and exposed to a 405 nm LED lamp at 28° with an intensity of 543 mW/cm^2^.

### 2.3. Free Radical Photopolymerization

Cu complexes/amine/Iodonium salt (0.2%/0.2%/1% *w*/*w*), gold (III) chloride (3 wt%), and silver nitrate (2 wt%) in DMF were added in small drops to make up the majority of the three-component photoinitiating systems. The PIS system was then dissolved in EGDA at a 95.6 weight percentage. To determine the weight percentage of the photoinitiating system, the monomer content was employed. The growth of the SPR nanoparticles was repeatedly screened using a Shimadzo UV-1800 spectrophotometer (Shimadzo, Duisburg, Germany). The polymer or composite was characterized by using TEM technique.

### 2.4. Redox Potentials

The oxidation and reduction potential (E_ox_, E_red_) in acetonitrile solution for the copper II complexes. It was estimated by quantifying the cyclic voltammetry with tetrabutylammonium hexafluorophosphate 0.1 M (TBAP) as a supporting electrolyte. The potential of the working electrode was gauged against the Ag/AgCl reference electrode (E^°^= 0.203 V versus standard hydrogen electrode (SHE)), a pure Pt wire was utilized as the counter electrode, and a platinum rod with a 0.2 cm^2^ surface area was utilized as the working electrode. The free energy change ∆G_et_ for the electron transfer reaction between the copper II complexes, diphenyl iodonium hexafluorophosphate (Iod,) and the amine (TEA) was determined using the classical Rehm–Weller equation (Equation (1)) [45,46].

∆G_et_ = E_ox_ − E_red_ − E* + C (1)

### 2.5. FTIR Experiments 

Jasco 6400 in the range 4000–400 cm^−1^ with a resolution of 4 cm^−1^ was used to measure FTIR.

### 2.6. Transmission Electron Microscopy (TEM) 

A field emission scanning electron microscope (JEM-100S, Joel, Tokyo, Japan) was used to examine the morphology and particle size of the polymer.

### 2.7. Fluorescence Experiments

A JASCO FP-8200 spectrometer (JASCO, Riyadh, Saudi Arabia) was used to determine the fluorescence properties of the copper complex in DMF.

### 2.8. Scanning Image Microscope (SEM)

The morphology and particle size of the polymer were examined by the Field Emission Scanning Electron Microscope (FESEM) (JEOL JSM 6490-A).

## 3. Results

### 3.1. Light Absorption of Cu-Complexes

**Figure 1:**
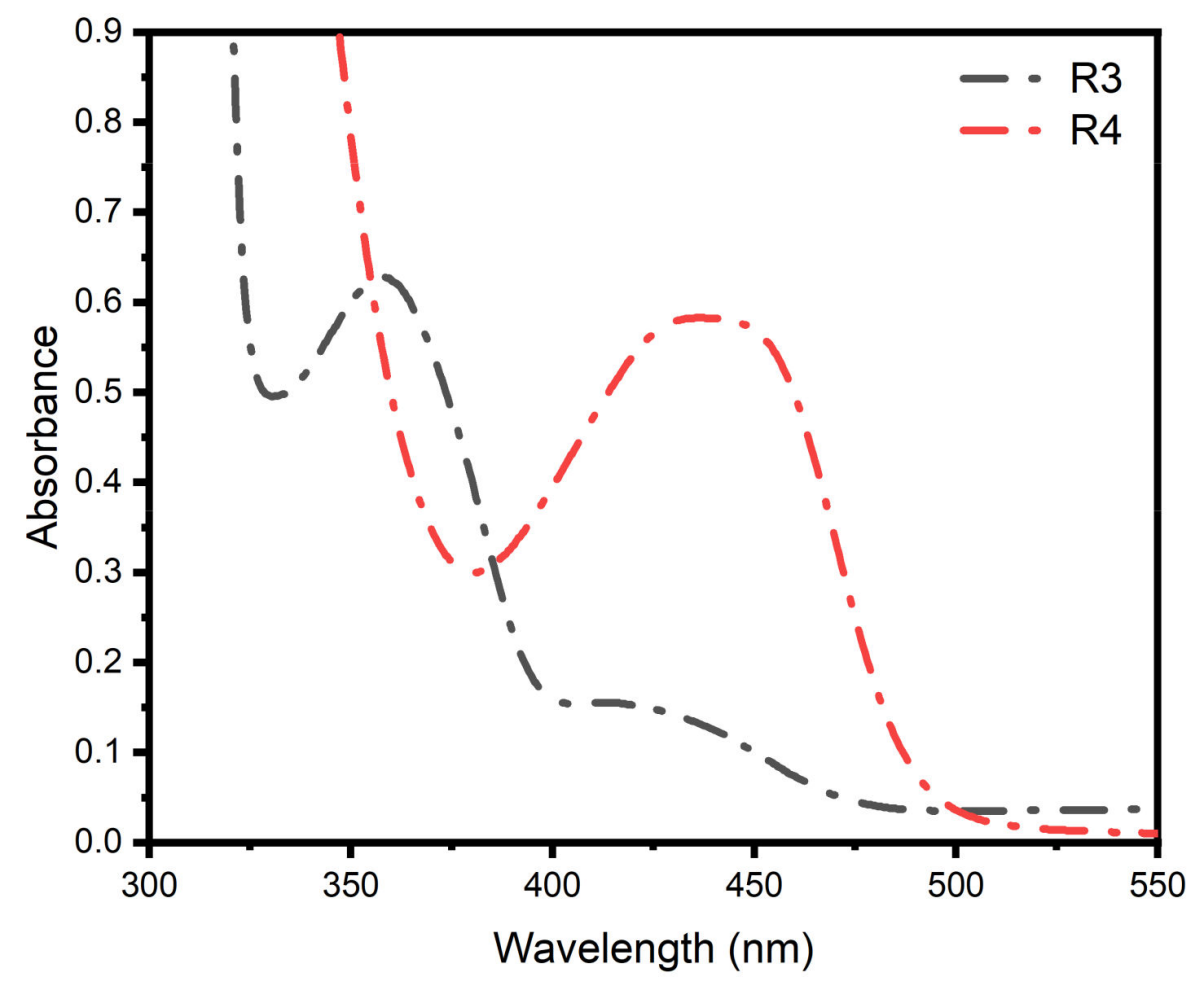
UV-Visible spectra R4 and R3 complexes in DMF concentration of 10^−4^ M.

The study of the UV-Vis. electronic spectra of the copper II complexes was carried out. It can be seen from Figure 1, that the examined complexes’ UV-Vis absorption spectra were attained in dimethylformamide at a concentration of 10^–4^ M. The solvent tested the main band of the investigated compound, which was situated in the spectral range of 438–429 nm. This band is representative of a ligand–metal charge transfer transition (LMCT) [30,47,48,49]. In the UV-Visible region noted from the reviewed compound in the system, the band is localized around 355–475 nm area with an extreme of 438 nm; this is allocated to the π→π* transition of the C=N [30,50]. The copper II complexes are categorized by high molar extinction coefficients (see Table 1).

### 3.2. Copper II Complex Oxidation Process

The free energy change of the Cu complexes/Iod salt electron transfer reaction is massively negative and makes the process favorable (using E_red_ (Iod) = −0.2 eV) [46]. The outcomes of the investigations on the photochemical mechanism of the initiation process would not be impacted by the counter anions of iodonium salts. For the reaction free energy of the copper II complexes and the triethylamine is ∆G_et_ = −1.28 eV for R3 and for R4 is ∆G_et_ = −1.58 eV, which also specifies a favorable electron transfer method (using E_ox_ = 1.079 V for the triethylamine) [30]. The results are summarized in Table 2. 

### 3.3. Photoinduced Synthesis of Au Nanoparticles:

**Figure 2:**
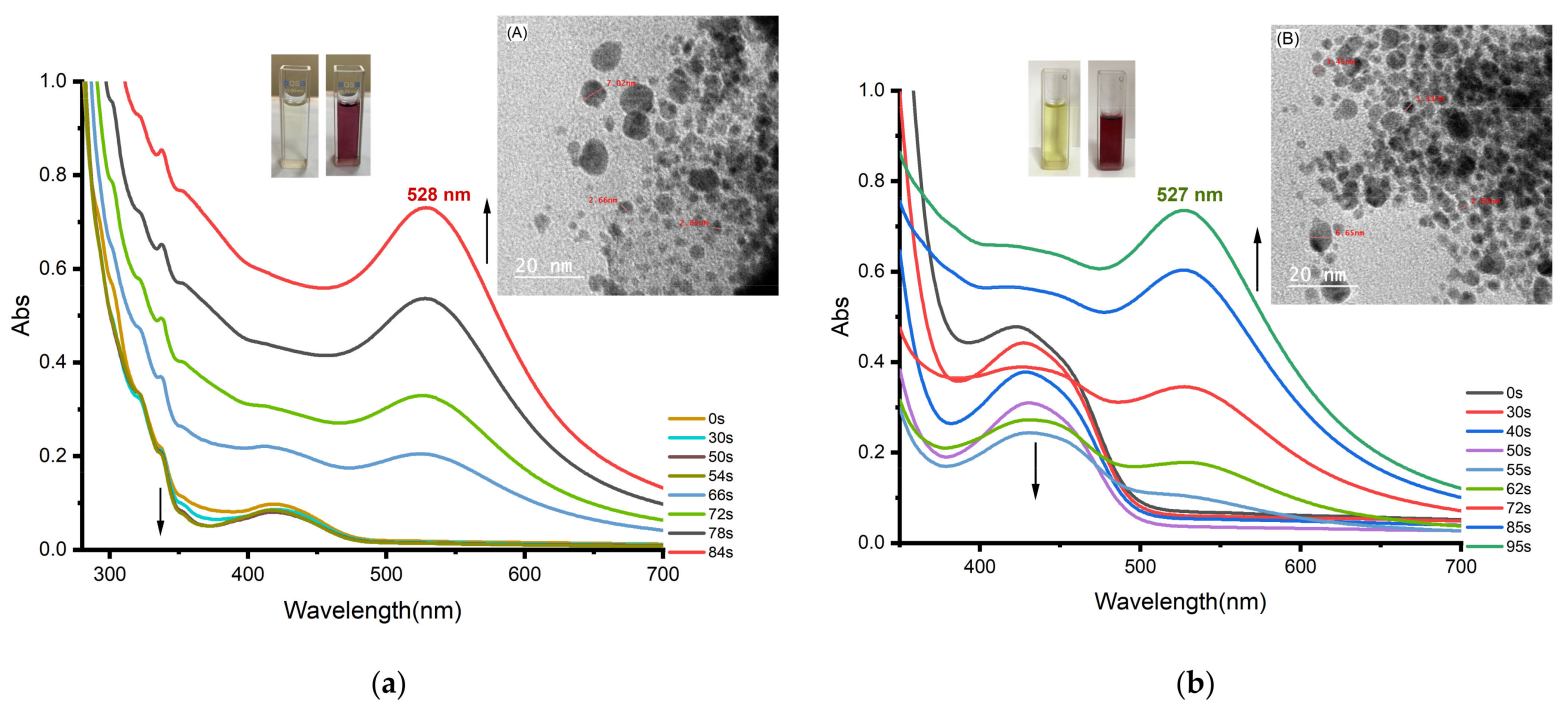
Evolution of the absorption spectra of the irradiated mixtures (λ_irr_ = 405 nm). Solution: (R3 (**a**) and R4 (**b**)) 1 × 10^−4^ M gold chloride 3 wt% dissolved in 25 mL of DMF. TEM micrographs of gold nanoparticles of the R3/gold chloride 3 wt% (**A**) and R4 (**B**).

Due to their Surface Plasmon Resonance (SPR) property, which is the collective oscillation of the conduction electrons in resonance with the wavelength of the irradiation light, noble metals are known to display distinctive optical properties. In the current investigation, UV-visible spectroscopy was used to first confirm the creation of gold nanoparticles by measuring the SPR peaks. Gold nanoparticles display size and shape-dependent plasmon absorption bands. We have studied different concentrations of the salts HAuCl_4_ (*w*/*w*) (1 wt%, 2 wt%, 3 wt%, and 5 wt%) to select the best concentration. Figure 2 displays the UV-Vis spectra of Au nanoparticles created in the presence of 1 mL (3 wt%) of gold III chloride and 2 mL (1 × 10^−4^ M) of (R3, R4) solution in DMF for the production of gold nanoparticles. During irradiation, the sample shows a reduction in absorption at 362, 445 nm, with the formation of a peak at 528, 527 nm. By using a LED at 405 nm, with an intensity of 543 mW/cm^2^ at 28 °C, the color progressively transitioned from yellow to dark purple in less than a minute (see Figure 2). Several indications have been observed for the formation of Au nanoparticles, namely the absorption at 528, 527 nm, the SPR peak, color change, and characteristics of the surface plasmon absorption spectra [30,51,52].

**Figure 3:**
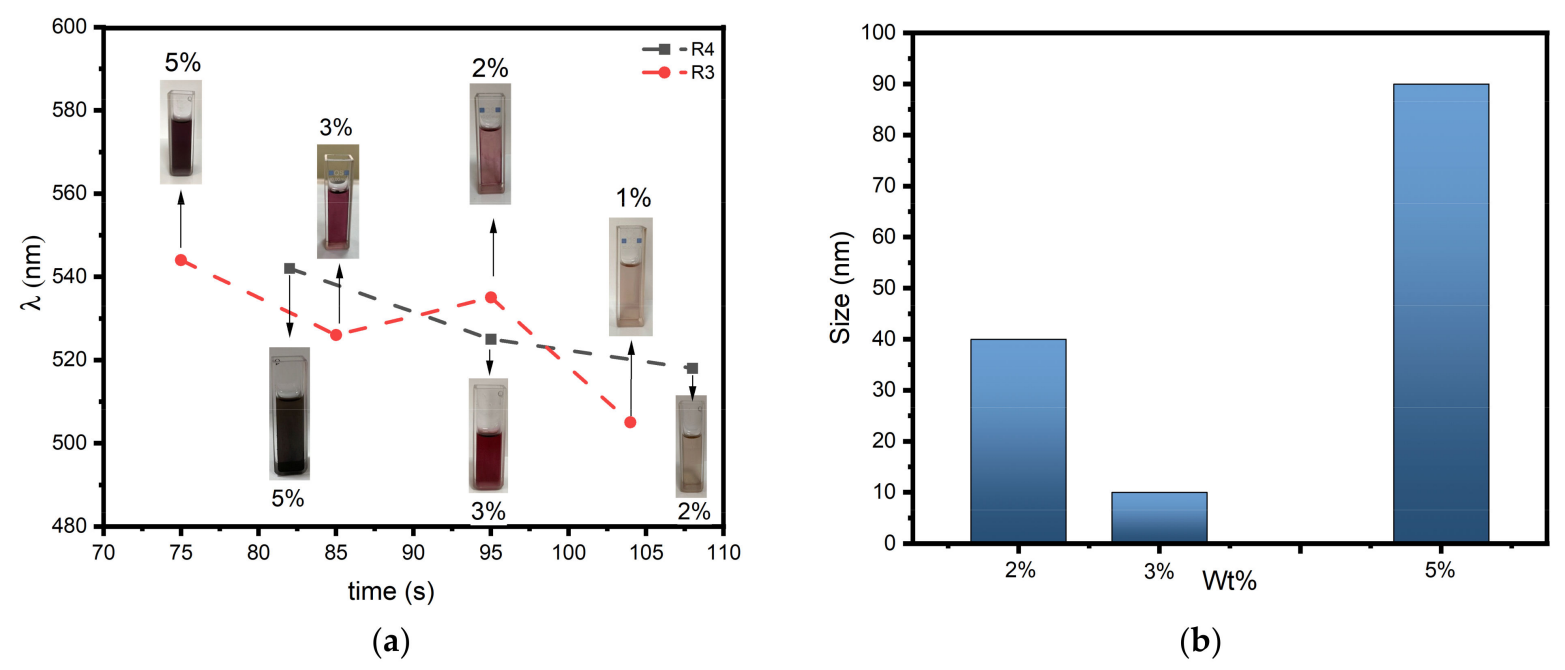
The salts HAuCl_4_ are prepared at different (*w*/*w*) (1%, 2%, 3%, 5%) (**a**) and comparison between different sizes of gold nanoparticles (**b**).

It was observed that the size, shape, composition, crystallinity, and structure of metal nanoparticles have a major role in determining their inherent qualities. The properties of these nanoparticles might theoretically be adjusted by changing any one of these parameters. Figure 2 illustrates how the color of such tiny particles can be systematically changed, varying from yellow at 55 s to pink and violet when it was allowed to coalesce in a controlled manner. In this study, we used the concentration of 3 wt% as depicted in (Figure 3). As you can see in Figure 3, the higher concentration 5 wt% R3 or R4 have a dark violet color and a large size of NPs produced in solution. The 3 wt% concentration was the correct concentration selected at the nanoparticle size level, which is why we chose it for all our studies. TEM images show the nanoparticle size ranging from 1–10 nm (see Figure 2a,b); this is a small size compared to other concentrations. It is noted that it occurs during the red shift of λ_max_ (nm) (see Figure 3). 

Because the intensity of the surface plasmon peak is exactly proportional to the density of the nanoparticles in solution, it is possible to ascribe the steady increase in this peak’s intensity with reaction time to the creation of gold nanoparticles [53]. In order to create gold nanoparticles, 0.2 wt% TEA was added to a solution of Complexes (R3, R4) in DMF and 1 mL (3 wt%) of gold chloride. At room temperature, the sample was continuously stirred for five minutes. During irradiation (LED at 405 nm with an intensity of 543 mW/cm^2^ at 28°C), the R3, R4/TEA/HAuCl_4_ systems show that the absorbance at 325 and 332 nm decreased, and a peak at 526 nm and 538 appeared (see Figure S2), respectively. Consequently, the color changes progressed from yellow to dark purple in less than a minute. The production of Au nanoparticles is indicated by the absorption in the 526–521 nm range corresponding to the SPR peak [30,51,52].

After one minute of exposure, we observed a significant decrease in the band at 338, 329 nm related to the disappearance of Au^+3^. Figure S3 shows the decrease of the R3 and R4 absorption during irradiation and the appearance of new peaks at 526 nm and 521, respectively. The linear correlation between the absorbance at 370, 407 nm vs. the range of 545–535 nm (inset in Figure S3) shows the declines in the absorption at 370–415 nm range during irradiation (R3, R4) with the concurrent increase of the surface plasmon band. The value of ∆G_et_ also demonstrates the potential for an electron transfer mechanism that might result in the creation of phenyl radical. These radicals have the ability to change Au^+3^ into Au^+2^. The Au^+2^ is unsteady. 

**Figure 4:**
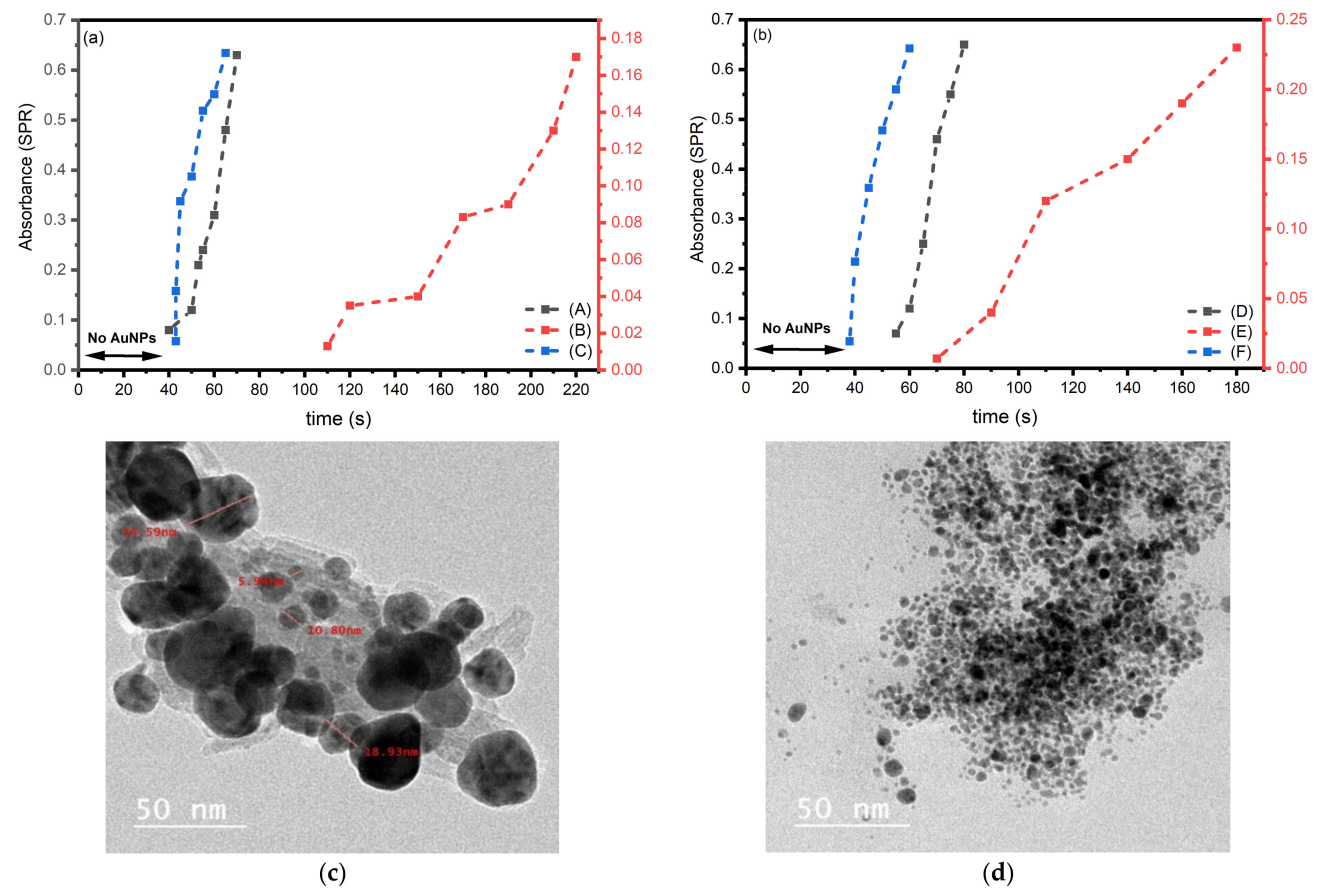
Plots of comparison between the PIS system based on R3 0.2 wt%/TEA 0.2 wt%/HAuCl_4_ 3 wt% (A), on R3 0.2 wt%/Iod 1 wt%/HAuCl_4_ 3 wt%(B) and the PIS system based on R3 0.2 wt%/TEA 0.2 wt%/Iod 1 wt%/HAuCl_4_ 3 wt% solution(C) (**a**), and the PIS system based on R4 0.2 wt%/TEA 0.2 wt%/HAuCl_4_ 3 wt% (D), on R4 0.2 wt%/Iod 1 wt%/HAuCl_4_ 3 wt%(E) and the PIS system based on R4 0.2 wt%/TEA 0.2 wt%/Iod 1 wt%/HAuCl_4_ 3 wt% solution (F) (**b**). TEM micrographs of gold nanoparticles of the PIS system based on R3 0.2 wt%/TEA 0.2 wt%/Iod 1 wt% solution (**c**) and the R4 0.2 wt%/TEA 0.2 wt%/Iod 1 wt% solution (**d**).

Figure S4 shows the UV-Vis absorption spectra of the PIS system based on R3–4/TEA/Iod in the presence of gold chloride. The PIS produces gold nanoparticles by reducing Au^3+^ to Au^0^, and the emergence of distinctive plasmons in the UV-Vis absorption spectra at 543 and 524 nm serves as evidence for this conclusion. The symmetrical shape of the peak shows that the gold nanoparticles in the organic phase have a consistent size distribution [30,54]. The insert in Figure S4 shows how the SPR band changes as the irradiation time increases. It was also observed that the absorption of R3–4 complexes and Au^+3^ disappeared during the irradiation time (Figure S4a,b). These findings demonstrate that copper II complexes can be reduced by iodonium salt with the rapid generation of initiating radicals and generate gold NPs without any unfavorable side reactions. The photoreduction of gold III to gold metallic by the three-component photoinitiating system was presented in Figure S5. The effectiveness of systems containing one, two, or three components (PIS) in the production of gold nanoparticles (AuNPs) was compared and is presented in Figure 4. This figure shows that the systems based on R3–4 complexes combined with triethylamine (TEA) have a reactivity similar to the system based on R3–4, TEA, and iod salt. The two-component system based on R3–4 and iod salt has lower reactivity and requires more irradiation time to produce AuNPs. The PIS three-component system looks to be more efficient when more radicals are produced [55]. In two-component systems containing both amine or Iod salt, the radicals formed from the amines are generally more easily oxidized by metal complexes than the radicals formed from the Iod salt. This is because the amine group is a better electron donor than the Iod salt, making the amine radical more reactive towards metal complexes. When an amine radical is formed in the presence of a metal complex, it can readily transfer an electron to the metal complex, resulting in the formation of an amine-metal complex. This reaction is often used in metal-mediated oxidations and polymerizations.

In contrast, the Iod salt is less reactive towards metal complexes because it is a poor electron donor. When a phenyl radical is formed in the presence of a metal complex, it is less likely to transfer an electron to the metal complex and form a phenyl-metal complex.

Therefore, in two-component systems containing both amines and phenyl groups, the radicals formed from the amines are generally much more easily oxidized by metal complexes than the phenyl radicals.

**Scheme 2:**
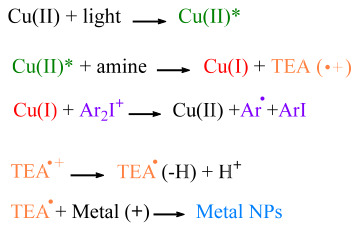
Proposal mechanism of the photoproduced Au/Ag NPS by the system based on R3-R4 (Cu II)/TEA (amine)/Iod salt (Ar_2_I^+^).

The only disadvantage observed for the three-component photoinitiator system (PIS) was the larger size of the gold nanoparticles (AuNPs), as seen in the transmission electron microscopy (TEM) images. The size of the AuNPs increased compared to the two-component system and a blueshift of approximately 6 nanometers (nm) in the λmax (nm) was observed (see Figure 4). Scheme 2 shows how the suggested reaction mechanism works. These findings show that a copper II complex can be reduced by simple irradiation in the presence of TEA and Iod salt with the quick formation of initiating radicals and metallic gold without any unfavorable side effects. 

The copper (II) complexes in the excited state can be reduced to copper (I) using a reducing agent such as triethylamine (TEA). This reduction can occur through a single-electron transfer mechanism, where the excited-state copper (II) complex donates an electron to TEA, resulting in the formation of copper (I) and a TEA radical cation.

In the presence of iodonium salts, copper (I) can react with iodonium ions to form copper (I) iodonium complexes, which are stable in solution. In addition, phenyl radicals can be generated through various methods, such as through the oxidation of phenyl groups by metal complexes or by the use of radical initiators.

The generated amine and phenyl radicals can then react with gold (III) to generate metallic gold nanoparticles (see Scheme 2). Aminoalkyl radicals are more easily oxidized.

### 3.4. Photoinduced Synthesis of Ag Nanoparticles

**Figure 5:**
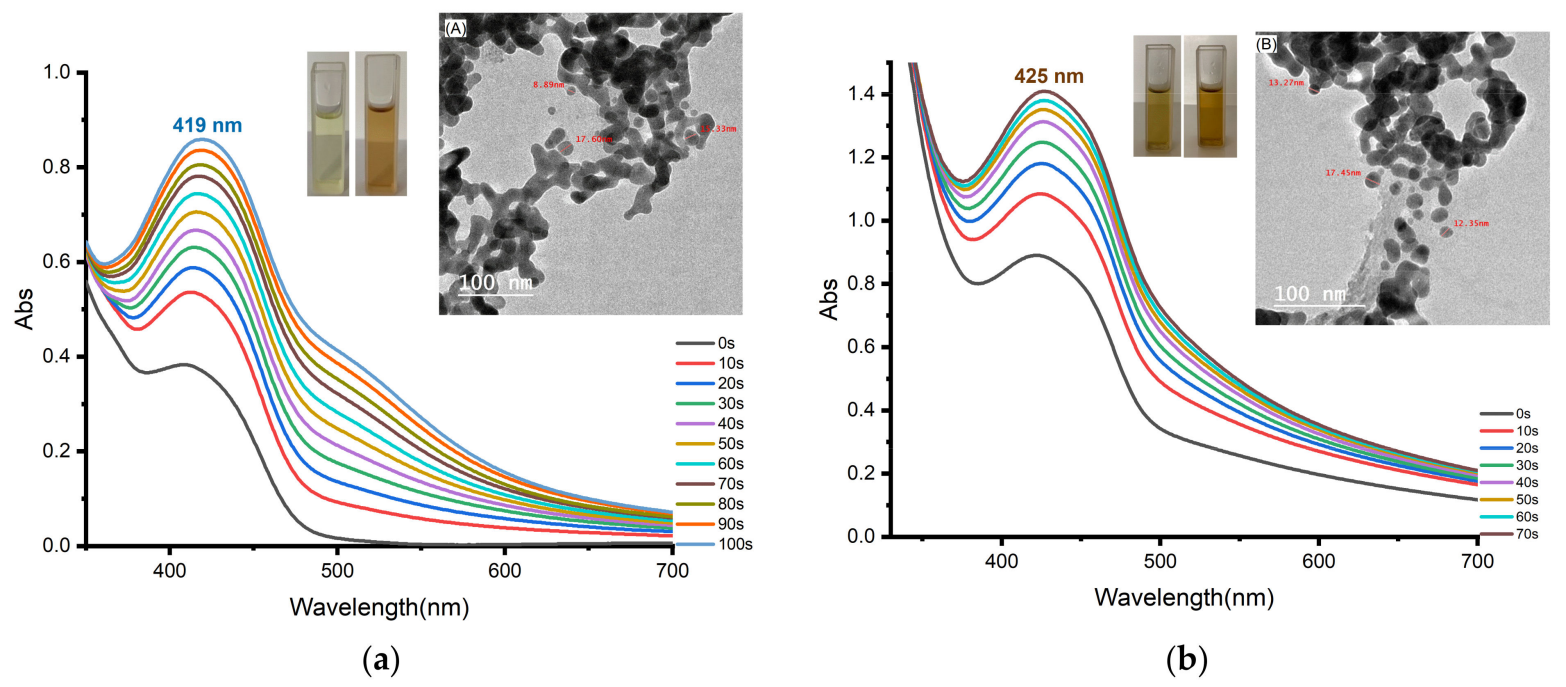
Evolution of the absorption spectra of the irradiated mixtures (λ_irr_ = 405 nm). Solution (R3 (**a**) and R4 (**b**)) 1 × 10^−4^ M AgNO_3_ 2 wt% dissolved in 25 mL of DMF. TEM micrographs of silver nanoparticles of the PIS system based on R3/AgNO_3_ 2 wt% (**A**) and R4 (**B**).

**Figure 6:**
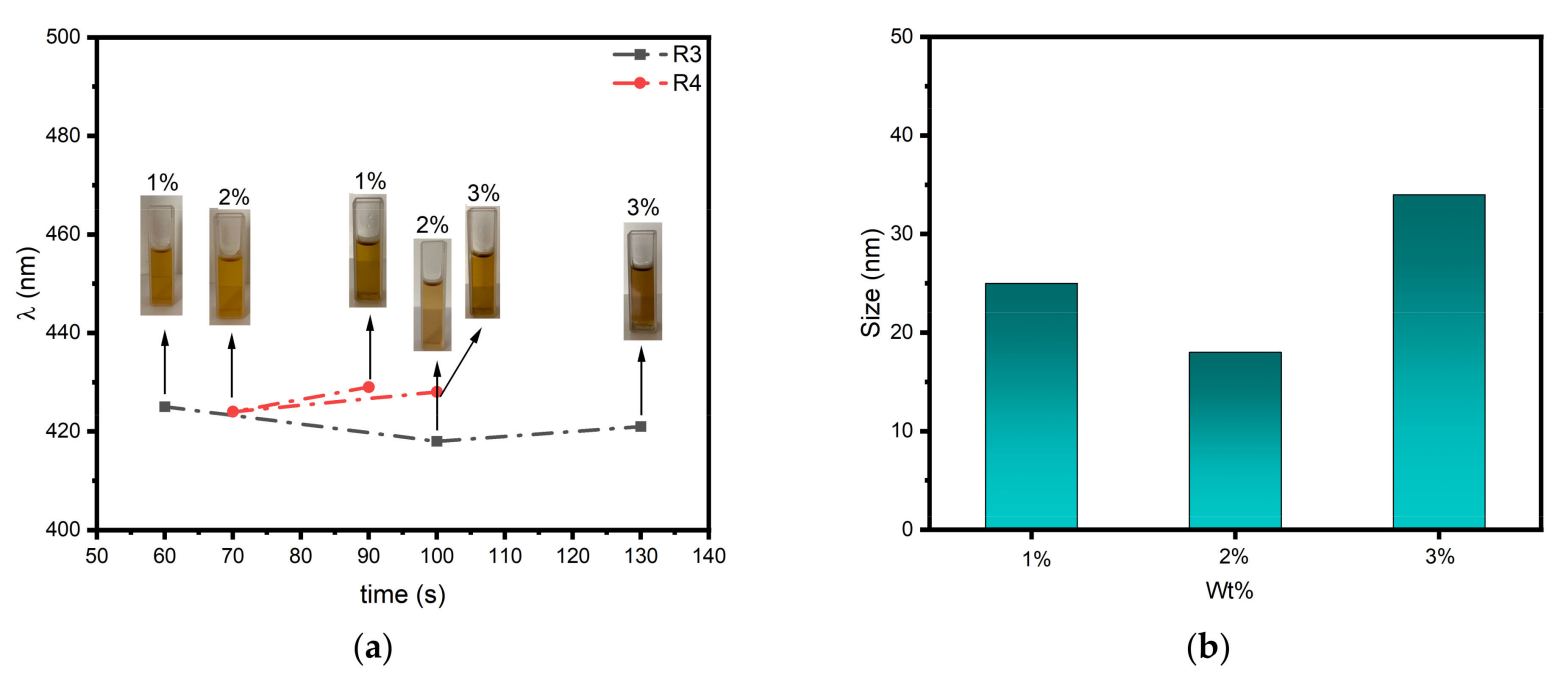
The salts AgNO_3_ are prepared at different (*w*/*w*) (1%, 2%, 3%) (**a**) and comparison between different sizes of silver nanoparticles (**b**).

Because of the appealing physicochemical characteristics of the AgNPs, they have attracted a lot of interest [56,57]. AgNPs are perfect for biotechnological applications since they also have the surface plasmon resonance (SPR) effect and great bacterial resistance to antibiotics [58]. In the current study, the formation of silver nanoparticles was first confirmed by measuring the SPR peaks using UV-Visible spectroscopy. Silver nanoparticles display size and shape-dependent plasmon absorption bands. The UV-Vis spectra of the Ag nanoparticles formed in the presence of 2 wt% AgNO_3_ (chosen as the best concentration of silver nitrate) are shown in Figure 5. Different concentrations of AgNO_3_ are studied to produce AgNPs (*w*/*w*) (1 wt%, 2 wt%, and 3 wt%) (see Figure 6). All solutions’ absorption spectra show a significant rise in the silver surface plasmon band (SP) in the 320–600 nm region [59]. Figure 5 includes photographic images of the relevant samples along with UV-Vis spectra that were collected before (0 sec) and after light exposure (up to 1 min). After briefly (less than 10 sec) exposing the photosensitive formulation LED@405 nm with an intensity of 543 mW/cm^2^ at 28 °C in atmospheric air, silver nanoparticles start to develop. 

As the illumination increases, the power of this peak’s creation of silver nanoparticles Ag^+^ increases, with a maximum exposure time of one minute [60]. As we previously indicated, it is now understood that the inherent properties of metal nanoparticles are primarily controlled by their size, shape, composition, crystallinity, and structure. In theory, each one of these variables might be changed to modify the characteristics of these nanoparticles [61]. Figure 6 illustrates how the color of AgNPs can be systematically changed from yellow to orange when such tiny particles are allowed to coalesce in a controlled way. In this investigation, we employed 2 wt% of the concentration of R3; larger concentrations resulted in darker orange hues and required longer processing times and longer wavelengths. TEM images show smaller nanoparticle sizes ranging from 1–20 nm for the 2 wt% concentration (see Figure 5a,b) and it is noted that it occurs during the red shift of λ_max_ (nm). In the lesser concentration, the time reaches a shorter period but with a very strong absorption peak, and for R4 it produces a very dark orange color. They investigated the impact of nanoparticle size on the aggregation process and showed that big nanoparticle size is the primary determinant that enables the rapid color shift in this sensing event. Figure 6 demonstrates that, in comparison to smaller silver nanoparticles, the rates of color change were progressively faster for bigger ones [61,62]. Figure S5 displays the UV-Vis spectra of the Ag nanoparticles produced in the presence of 1 mL of AgNO_3_ (2 wt%) and 0.2 mL of TEA 0.2 wt%. The complexes’ characteristic peaks at 442 and 417 nm absorb significantly more light when amine-based systems are used, and R3 and R4 dark green exhibit a change in color to green.

**Figure 7:**
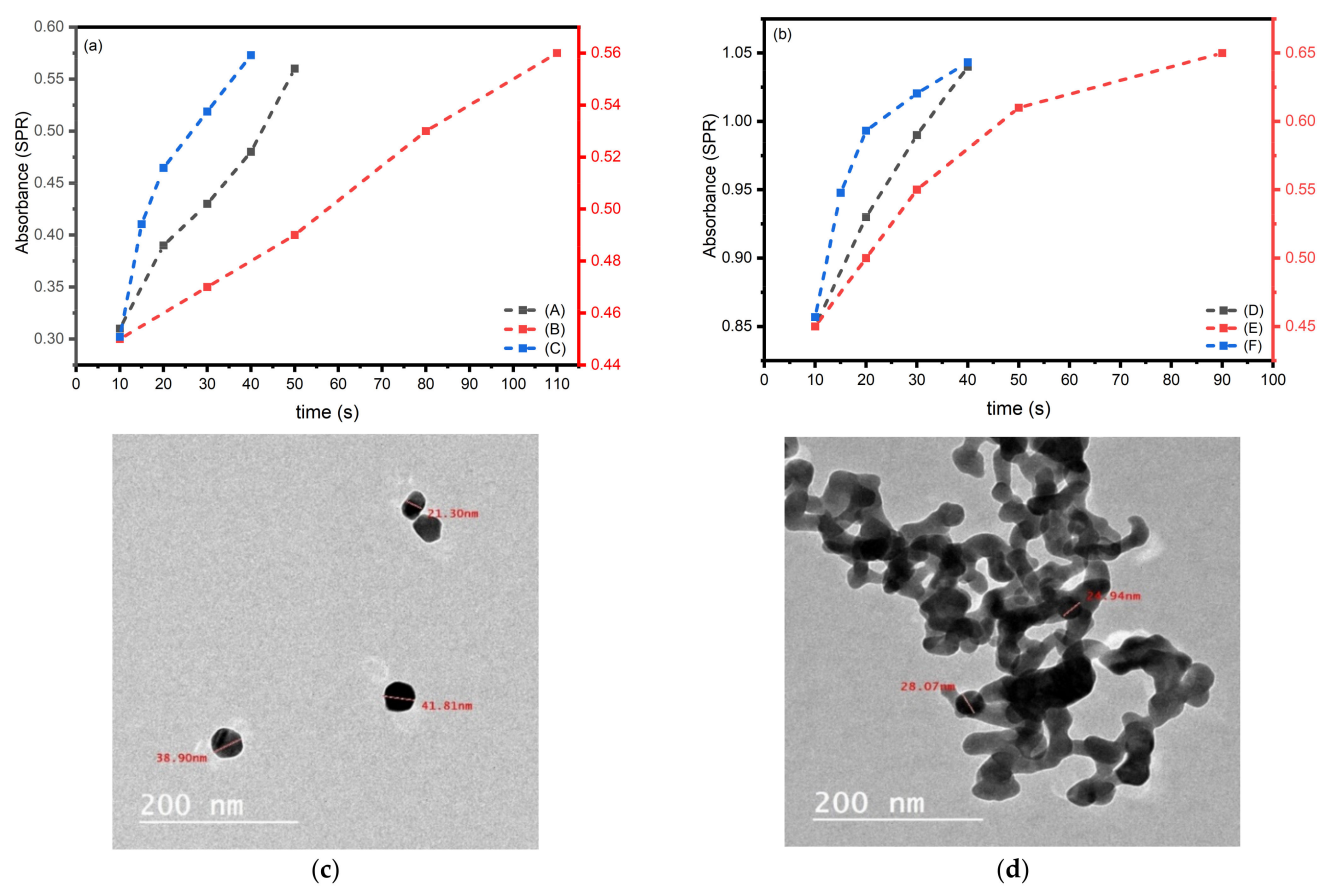
Plots of comparison between the PIS system based on R3 0.2 wt%/TEA 0.2 wt%/AgNO_3_ 2 wt% (A), on R3 0.2 wt%/Iod 1 wt%/AgNO_3_ 2 wt% (B) and the PIS system based on R3 0.2 wt%/TEA/Iod 1 wt%/AgNO_3_ 2 wt% solution (C) (**a**), and the PIS system based on R4 0.2 wt%/TEA 0.2 wt%/AgNO_3_ 2 wt% (D), on R4 0.2 wt%/Iod 1 wt%/AgNO_3_ 2 wt%(E) and the PIS system based on R4 0.2 wt%/0.2 wt%TEA 0.2 wt%/Iod 1 wt%/AgNO_3_ 2 wt% solution (F) (**b**). TEM micrographs of gold nanoparticles of the PIS system based on R3/TEA/Iod AgNO_3_ 2 wt% solution (**c**) and the R4 0.2 wt%/TEA 0.2 wt%/Iod 1 wt%/AgNO_3_ 2 wt% solution (**d**).

The development of Ag nanoparticles is indicated by the alteration in color and dis-tincture surface plasmon absorption spectra [63,64,65,66]. It was discovered that the addition of TEA boosted the reduction rate of Ag^+^ ions, which in turn increased the nucleation and growth rates of Ag particles. This demonstrates TEA involvement in the acceleration of the reduction reaction. Figure S5 displays the UV-Vis spectra of Ag nanoparticles created in the presence of 1 mL of AgNO_3_ and 0.2 mL of TEA. Light irradiation with an LED at 405 nm with an intensity of 543 mW/cm^2^ at 28 °C significantly boosts absorption of the complexes’ characteristic peak near 442 and 417 nm, and the color shift to green for R3 and R4 dark green in complex/amine-based systems. Instantaneous changes in the colors of the combined solutions may be plainly noticed visually. The system light irradiation during the photolysis process for the (R3–4/iodonium salt/amine) was extended to 200 s due to the slow response rate of the complexes and iodonium salt (see Figure S6). The Ag^+^ can then be reduced by another radical to Ag (0). Similar to the three-component PIS, interactions in the Cu complexes (R3–4)/Iod/amine-based PISs were carried out, and their UV-visible absorption spectra are shown in Figure S7. The absorption of the peak at about 410 and 433 nm is significantly increased within 40 s. The PIS system Cu complexes (R3–4)/TEA/Iod solution UV-Vis absorption spectra obtained during irradiation in the presence of AgNO_3_ are shown in Figure S7. Noting the efficiency of the three-component PIS system in relation to the two-component PIS relative to the reaction time in the ternary system is higher with the increase in the size of the nanoparticles (see Figure 7). The development of distinctive plasmons in the UV-Vis absorption spectra at 400 nm serves as evidence that the reduction of Ag^+^ to Ag^0^ caused the synthesis of silver nanoparticles in the PIS [53]. Scheme 2 shows how the suggested reaction mechanism works.

### 3.5. Fabrication of AuNPs and AgNPs Embedded Polymer

After studying the R3–4 Complexes/Iod/TEA (0.2 wt%/1 wt%/0.2 wt%) three-component photoinitiating system, we found it to be an efficient system for free radical polymerization. Furthermore, we successfully prepared and stabilized nanoparticles in situ using a one-step method, which is a convenient and effective approach for producing stable nanoparticles.

**Figure 8:**
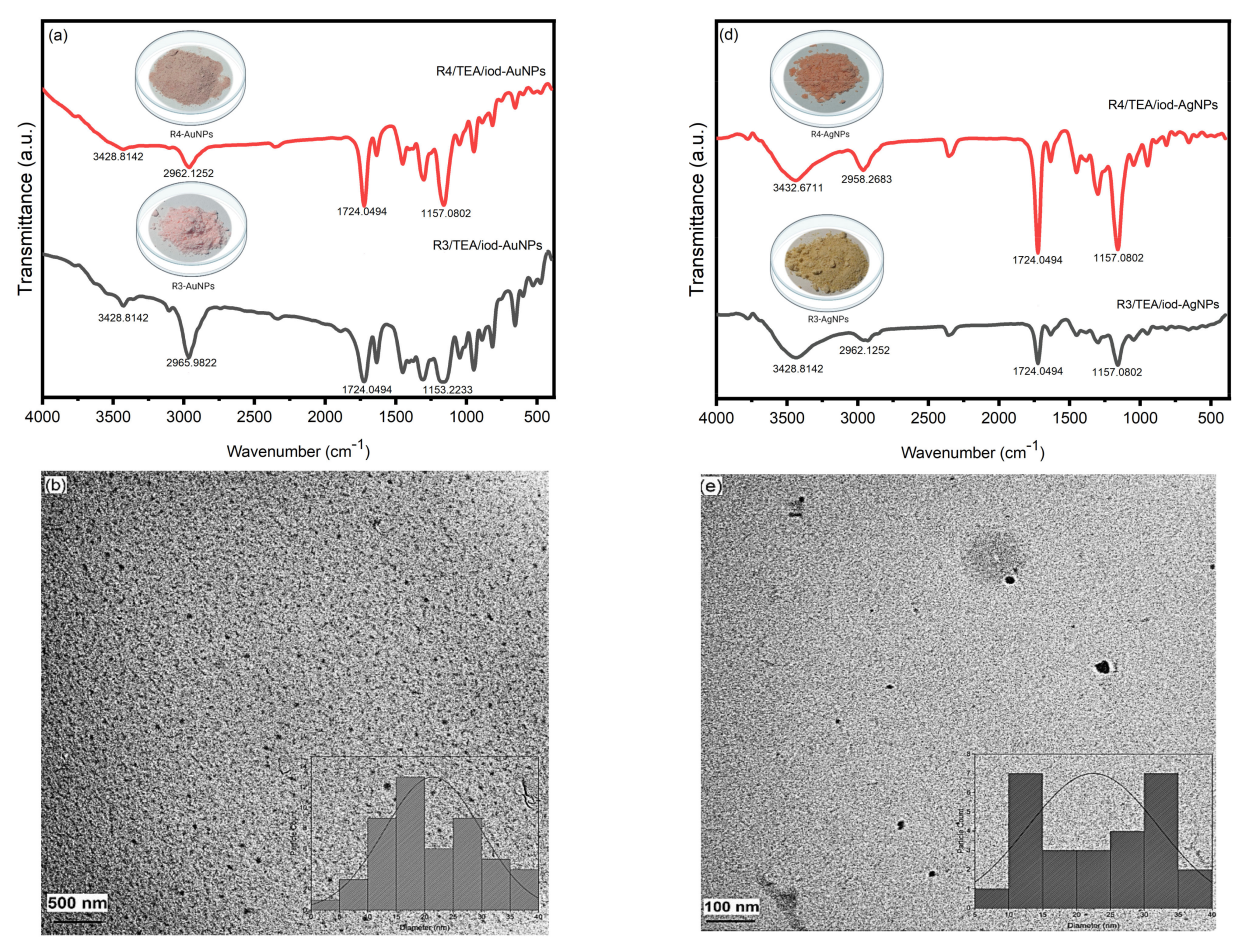

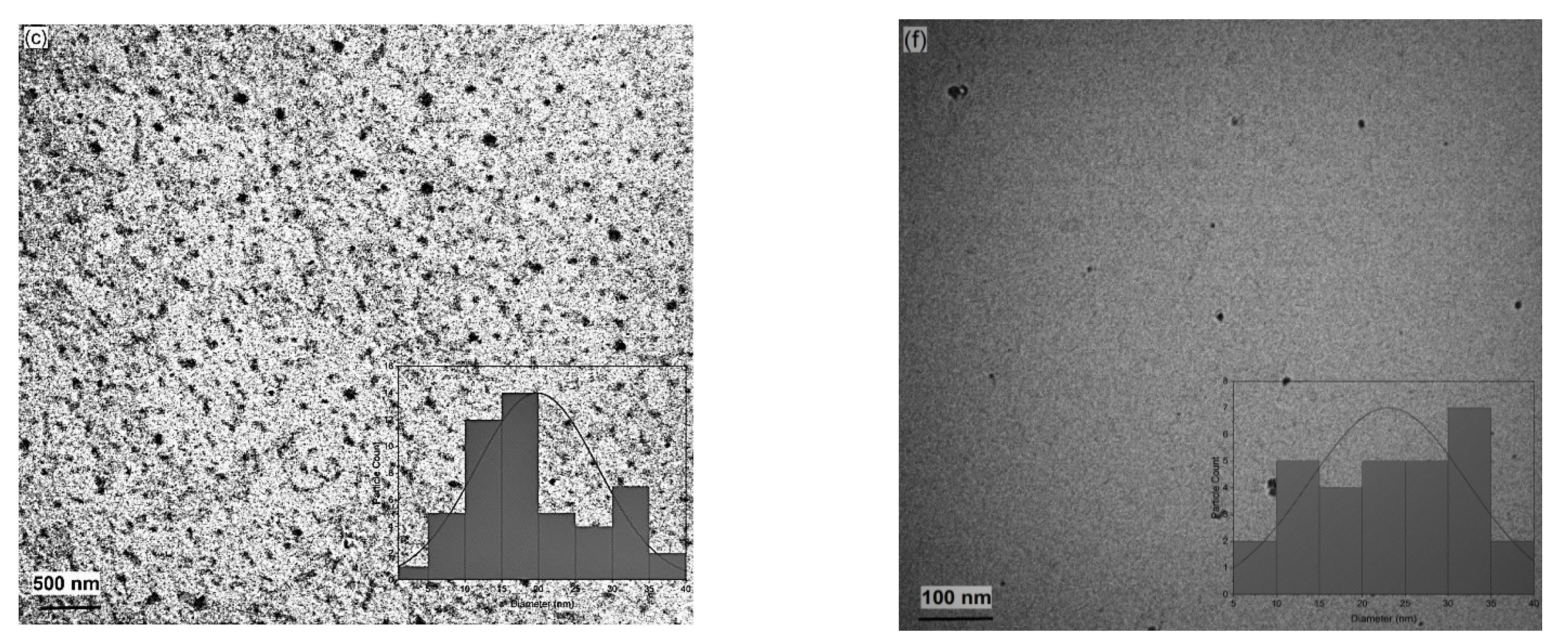
FT-IR spectrum of (R3 and R4) Complexes, AuNPs and AgNPs with Iod 1 wt%, TEA 0.2 wt%, (**a**) is R3–4 AuNPs and (**d**) is R3–4 AgNPs. TEM image of gold and silver nanoparticles in the polymer matrix complexes R4 and R3 0.2 wt%. (**b**) is R3(AuNPs+Iod), (**c**) R4(AuNPs+Iod), (**e**) R4(AgNPs+Iod)), (**f**) R3(AgNPs+Iod). AgNO_3_ 2 wt%, gold chloride 3 wt%, and TEA 0.2 wt% dissolved in EGDA 95.6 wt%.

**Figure 9:**
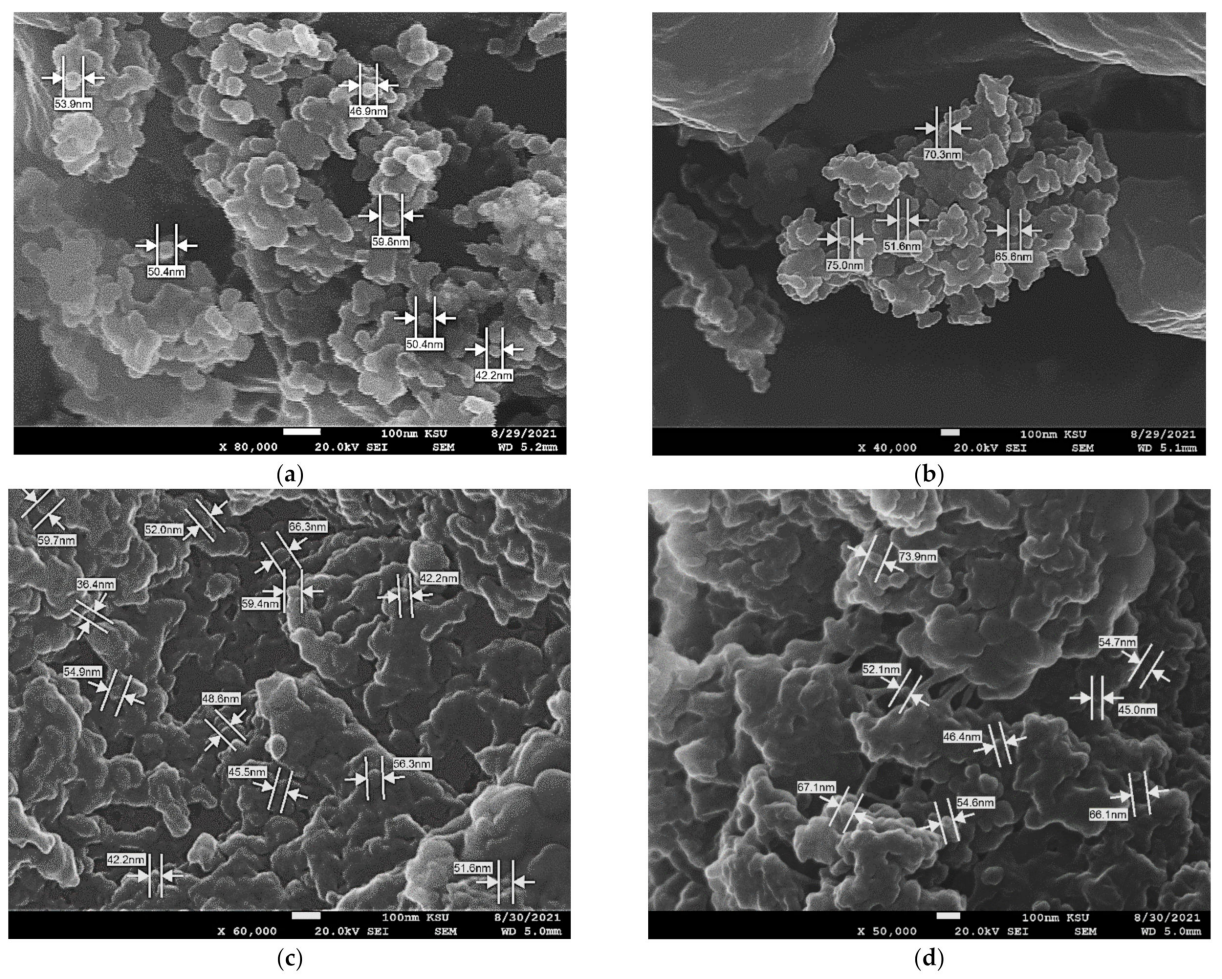
SEM image of (R3 and R4) Complexes, AuNPs and AgNPs with Iod 1 w% (**a**) is R4-AuNPs and (**b**) is R3-AuNPs (**c**) is R4-AgNPs (**d**) is R3-AgNPs Iod 1 w% AgNO_3_ 2 wt%, gold chloride 3 wt%, and TEA 0.2 wt% dissolved in EGDA 95.6 wt%.

Nanoparticle preparation and stabilization can be accomplished in situ in one step. This method offers the chance to create stabilized NPs in a quick and simple manner. UV-Vis spectroscopic examinations of the UV-crosslinked samples that were irradiated for 25 min in the air were performed. Cu complexes (R3–4), Iod, and TEA serve as the photoinitiating system in the sample, which also contains EGDA as a monomer in the presence of gold chloride. R3–4 0.2 wt%, gold (III) chloride 3 wt%, or silver nitrate 2 wt%, TEA 0.2 wt%, and iodonium salt 1 wt% dissolved in EGDA at 95.6 wt% were used to create the photopolymerization formulation. The last ingredient was continuously stirred at room temperature. The formation of plasmon resonance gold nanoparticles, as was seen for the UV irradiation of a photoinitiating system containing the gold and silver ion, can be attributed to the significant absorption peak shown (see Figure S8) at 532 and 552 nm. The creation of the AuNPs SPR band amply demonstrates the UV-induced synthesis of Au nanoparticles in the cross-linked acrylate formulation. Another clear indicator that the AuNPs are developing and depositing inside the polymeric template is the color change of these samples from yellow to purple [30,67]. The cross-linked acrylate formulation embedded Au nanoparticles is clearly confirmed by the development of an SPR band of AuNPs. This powder color alterations from yellow to light purple are more evidence in favor of the creation of AuNPs (see Figure 8a). The FT-IR spectra of photopolymerization reaction (see Figure 8a) demonstrate that they were located between 3779 and 3429 cm^−1^ and that the reduction of Au^3+^ to Au^0^ produces prominent bands at 3430 cm^−1^ and 2922 cm^−1^ [68,69]. The absorption peak at 1724 cm^−1^ exhibited in the spectra is allocated to C=O stretching in EGDA [70]. It has been reported that the FT-IR spectrum of silver nanoparticles (AgNPs) includes bands at 2950 cm^−1^ and ~3400 cm^−1^ in the region corresponding to –CH_2_ stretching vibration, respectively [71] (see Figure 8d). SEM and TEM measurements, along with Figure 8b,c,e,f and Figure 9 were used to validate the regulated size distribution of AgNPs and AuNPs. The majority of the nanoparticles have spherical or disk-like shapes, with diameters ranging from 5 to 40 nm for both gold and silver particles. The ethylene glycol-based acrylic monomer’s chelation effect facilitated the size, shape, and dispersion of the NPs [72]. The powder obtained confirmed that a high degree of crosslinking was obtained. 

## 4. Conclusions

This study proposes the use of a novel copper(II) complexes (R3–4) based on a benzimidazole Schiff base as a photocatalyst for the polymerization of ethylene glycol diacrylate and for the production of gold and silver nanoparticles within a polymer network under LED irradiation at 405 nm with an intensity of 543 mW/cm^2^ at 28 °C. Scanning electron microscope (SEM) analysis showed that composites with nanoparticle sizes of 5–40 nm were formed after brief irradiation of a mixture containing the copper (II) complex, triethylamine (TEA), and iodine salt with polymerizable acrylate groups (EGDA). The gold and silver nanoparticles were found to be spherical and evenly dispersed.

Based on the study of photoinitiating systems in solution, it was observed that both complexes R3 and R4 showed the efficient and rapid formation of nanoparticles, with a size range of 1–10 nm and a formation time of approximately 84 s in a one-component system. The reactivity of the two-component system based on R3–4/TEA was similar to that of the three-component system based on R3–4/TEA/Iod salt, with high efficacy in producing nanoparticles due to the more effective amine radicals formed by the reduction of copper complexes. Systems consisting of R3–4/Iod salt were found to be less efficient than the amine-containing system, taking much longer.

## Figures and Tables

**Table 1:** Molar extinction coefficients ε in different solvents for the Cu-complexes investigated.

Complexes	Solvent	λ_max_ (nm)	ε (M^−1^ cm^−1^)
**(R3)**	DMF	355	6220
**(R4)**	DMF	438	5820

**Table 2:** Excited state energies E*, oxidation potentials E_ox_, and free energy change (∆G_et_) for the Cu complexes/amine/Iod interaction.

E_ox_ vs. SCE* [V] (Cu Complexes)	E_red_ vs. SCE* [V]	E*[eV]	∆G_et_ (Cu Complexes/TEA) (eV)	∆G_et_ (Cu Complexes/Iod) (eV)
(R3) 1.01	−0.59	2.95	−1.28	−1.74
(R4) 1.05	−0.47	3.13	−1.58	−1.88

## Data Availability

Not applicable.

## Supplementary Materials

The following supporting information can be downloaded at: https://www.mdpi.com/article/10.3390/polym15051289/s1, Scheme S1. Synthesis of Ligands HL1 (1) and HL2 (2); Scheme S2. Structure of mixed complexes (3) and (4); Table S1. Analytical data of mixed synthesized complexes; Table S2. The vibrational assignment wavenumbers in cm^−1^ of ligands and mixed complexes; Table S3. X-ray powder diffraction crystal data of complexes: Lattice constant, inter axial angle, Crystal system, unit cell volume, 2θ range and cristallinity size of different metal complex; Figure S1. Cyclic voltammogram of Cu complexes in Acetonitrile (a) R4 complex and (b) R3 complex; Figure S2. Luminescence spectra of R3 (a), and R4 (b) complexes in DMF concentration of 10^−4^ M; Figure S3. Evolution of the absorption spectra of the irradiated mixtures (λ_irr_ = 405 nm). Solution: (R3 (a) and R4 (b)) 1 × 10^−4^ M gold chloride 3 wt% and TEA 0.2 wt% dissolved in 25 mL of DMF; Figure S4. Evolution of the absorption spectra of the irradiated mixtures (λ_irr_ = 405 nm). Solution: (R3 (a) and R4 (b)) 1 × 10^−4^ M gold chloride 3 wt% and iodonium salt 1 wt% dissolved in 25 mL of DMF; Figure S5. Evolution of the absorption spectra of the irradiated mixtures (λ_irr_ = 405 nm). Solution: (R3 (a) and R4 (b)) 1 × 10^−4^ M gold chloride 3 wt%, TEA 0.2 wt%, iodonium salt 1 wt% dissolved in 25 mL of DMF; Figure S6. Evolution of the absorption spectra of the irradiated mixtures (λ_irr_ = 405 nm). Solution (R3 (a) and R4 (b)) 1 × 10^−4^ M AgNO_3_ 2 wt% and TEA 0.2 wt% dissolved in 25 mL of DMF; Figure S7. Evolution of the absorption spectra of the irradiated mixtures (λ_irr_ = 405 nm). Solution (R3 (a) and R4 (b)) 1 × 10^−4^ M AgNO_3_ 2 wt% iodonium salt 1 wt% dissolved in 25 mL of DMF; Figure S8. Evolution of the absorption spectra of the irradiated mixtures (λ_irr_ = 405 nm). Solution (R3 (a) and R4 (b)) 1 × 10^−4^ M AgNO_3_ 2 wt%, iodonium salt 1 wt%, and TEA 0.2 wt% dissolved in 25 mL of DMF; Figure S9. Time evolution of the absorption spectrum of the ethylene glycol diacrylate (EGDA) during photopolymerization, irradiated mixtures (λ_irr_ = 405 nm). Solution: R3 in (a) and R4 in (b) 1 × 10^−4^ M gold chloride 3 wt%, TEA 0.2 wt%, and iodonium salt 1 wt% dissolved in 95.6 wt%. References [73,74,75,76,77,78] are cited in the supplementary materials.

## References

[1] Pandey S., Mishra S.B. (2011). Sol–gel derived organic–inorganic hybrid materials: Synthesis, characterizations and applications. J. Sol-Gel Sci. Technol..

[2] Zhang J.Z. (1997). Ultrafast Studies of Electron Dynamics in Semiconductor and Metal Colloidal Nanoparticles: Effects of Size and Surface. Acc. Chem. Res..

[3] Thomas J.M. (1988). Colloidal metals: Past, present and future. Pure Appl. Chem..

[4] Perenboom J., Wyder P., Meier F. (1981). Electronic properties of small metallic particles. Phys. Rep..

[5] Sun Y., Xia Y. (2002). Shape-Controlled Synthesis of Gold and Silver Nanoparticles. Science.

[6] Shanmugam S., Viswanathan B., Varadarajan T.K. (2007). Photochemically reduced polyoxometalate assisted generation of silver and gold nanoparticles in composite films: A single step route. Nanoscale Res. Lett..

[7] Zhou X., Zhao Z., He Y., Ye Y., Zhou J., Zhang J., Ouyang Q., Tang B., Wang X. (2018). Photoinduced synthesis of gold nanoparticle–bacterial cellulose nanocomposite and its application for in-situ detection of trace concentration of dyes in textile and paper. Cellulose.

[8] Batibay G.S., Gunkara O.T., Ocal N., Arsu N. (2018). In-situ photoinduced formation of self–assembled Ag NPs using POSS-TX as nano-photoinitiator in PEGMEA/PEGDA polymer matrix and creating self-wrinkled pattern. J. Photochem. Photobiol. A Chem..

[9] Balan L., Jin M., Malval J.-P., Chaumeil H., Defoin A., Vidal L. (2008). Fabrication of Silver Nanoparticle-Embedded Polymer Promoted by Combined Photochemical Properties of a 2,7-Diaminofluorene Derivative Dye. Macromolecules.

[10] El-Sayed M.A. (2001). Some Interesting Properties of Metals Confined in Time and Nanometer Space of Different Shapes. Acc. Chem. Res..

[11] Eustis S., El-Sayed M.A. (2006). Why gold nanoparticles are more precious than pretty gold: Noble metal surface plasmon resonance and its enhancement of the radiative and nonradiative properties of nanocrystals of different shapes. Chem. Soc. Rev..

[12] Balan L., Malval J.-P., Schneider R., Burget D. (2007). Silver nanoparticles: New synthesis, characterization and photophysical properties. Mater. Chem. Phys..

[13] Kong H., Jang J. (2006). One-step fabrication of silver nanoparticle embedded polymer nanofibers by radical-mediated dispersion polymerization. Chem. Commun..

[14] Scaiano J.C., Billone P., Gonzalez C.M., Marett L., Marin M.L., McGilvray K.L., Yuan N. (2009). Photochemical routes to silver and gold nanoparticles. Pure Appl. Chem..

[15] Korchev A.S., Bozack M.J., Slaten B.L., Mills G. (2003). Polymer-Initiated Photogeneration of Silver Nanoparticles in SPEEK/PVA Films: Direct Metal Photopatterning. J. Am. Chem. Soc..

[16] Balan L., Melinte V., Buruiana T., Schneider R., Vidal L. (2012). Controlling the morphology of gold nanoparticles synthesized photochemically in a polymer matrix through photonic parameters. Nanotechnology.

[17] Sangermano M., Yagci Y., Rizza G. (2007). In Situ Synthesis of Silver−Epoxy Nanocomposites by Photoinduced Electron Transfer and Cationic Polymerization Processes. Macromolecules.

[18] Sudeep P.K., Kamat P.V. (2005). Photosensitized Growth of Silver Nanoparticles under Visible Light Irradiation: A Mechanistic Investigation. Chem. Mater..

[19] Yagci Y., Sangermano M., Rizza G. (2008). A visible light photochemical route to silver–epoxy nanocomposites by simultaneous polymerization–reduction approach. Polymer.

[20] Balan L., Malval J.-P., Schneider R., Le Nouen D., Lougnot D.-J. (2010). In-situ fabrication of polyacrylate–silver nanocomposite through photoinduced tandem reactions involving eosin dye. Polymer.

[21] Giuffrida S., Costanzo L.L., Ventimiglia G., Bongiorno C. (2008). Photochemical synthesis of copper nanoparticles incorporated in poly(vinyl pyrrolidone). J. Nanoparticle Res..

[22] Mitzscherling S., Cui Q., Koopman W., Bargheer M. (2015). Dielectric function of two-phase colloid–polymer nanocomposite. Phys. Chem. Chem. Phys..

[23] Tian Z.-Q., Ren B., Wu D.-Y. (2002). Surface-Enhanced Raman Scattering: From Noble to Transition Metals and from Rough Surfaces to Ordered Nanostructures. J. Phys. Chem. B.

[24] Pacioni N.L., Pardoe A., McGilvray K.L., Chrétien M.N., Scaiano J.C. (2010). Synthesis of copper nanoparticles mediated by photogenerated free radicals: Catalytic role of chloride anions. Photochem. Photobiol. Sci..

[25] McGilvray K.L., Decan M.R., Wang D., Scaiano J.C. (2006). Facile Photochemical Synthesis of Unprotected Aqueous Gold Nanoparticles. J. Am. Chem. Soc..

[26] Maretti L., Billone P.S., Liu Y., Scaiano J.C. (2009). Facile Photochemical Synthesis and Characterization of Highly Fluorescent Silver Nanoparticles. J. Am. Chem. Soc..

[27] Scaiano J.C., Stamplecoskie K.G., Hallett-Tapley G.L. (2012). Photochemical Norrish type I reaction as a tool for metal nanoparticle synthesis: Importance of proton coupled electron transfer. Chem. Commun..

[28] Marin M.L., McGilvray K.L., Scaiano J.C. (2008). Photochemical Strategies for the Synthesis of Gold Nanoparticles from Au(III) and Au(I) Using Photoinduced Free Radical Generation. J. Am. Chem. Soc..

[29] Çeper T., Arsu N. (2017). Photochemically Prepared Gold/Polymer Nanocoatings: Formation of Gold Mirror. Macromol. Chem. Phys..

[30] Tar H., Kashar T.I., Kouki N., Aldawas R., Graff B., Lalevée J. (2020). Novel Copper Photoredox Catalysts for Polymerization: An In Situ Synthesis of Metal Nanoparticles. Polymers.

[31] Lalevée J., Blanchard N., Tehfe M.-A., Peter M., Morlet-Savary F., Fouassier J.P. (2011). A Novel Photopolymerization Initiating System Based on an Iridium Complex Photocatalyst. Macromol. Rapid Commun..

[32] Tehfe M.-A., Lepeltier M., Dumur F., Gigmes D., Fouassier J.-P., Lalevée J. (2017). Structural Effects in the Iridium Complex Series: Photoredox Catalysis and Photoinitiation of Polymerization Reactions under Visible Lights. Macromol. Chem. Phys..

[33] Tehfe M.-A., Lalevée J., Telitel S., Sun J., Zhao J., Graff B., Morlet-Savary F., Fouassier J.-P. (2012). Iridium complexes incorporating coumarin moiety as catalyst photoinitiators: Towards household green LED bulb and halogen lamp irradiation. Polymer.

[34] Tehfe M.-A., Dumur F., Telitel S., Gigmes D., Contal E., Bertin D., Morlet-Savary F., Graff B., Fouassier J.-P., Lalevée J. (2013). Zinc-based metal complexes as new photocatalysts in polymerization initiating systems. Eur. Polym. J..

[35] Xiao P., Dumur F., Zhang J., Fouassier J.P., Gigmes D., Lalevée J. (2014). Copper Complexes in Radical Photoinitiating Systems: Applications to Free Radical and Cationic Polymerization upon Visible LEDs. Macromolecules.

[36] Al Mousawi A., Kermagoret A., Versace D.-L., Toufaily J., Hamieh T., Graff B., Dumur F., Gigmes D., Fouassier J.P., Lalevée J. (2016). Copper photoredox catalysts for polymerization upon near UV or visible light: Structure/reactivity/efficiency relationships and use in LED projector 3D printing resins. Polym. Chem..

[37] Cuttell D.G., Kuang S.-M., Fanwick P.E., McMillin D.R., Walton R.A. (2001). Simple Cu(I) Complexes with Unprecedented Excited-State Lifetimes. J. Am. Chem. Soc..

[38] Zhang Q., Zhou Q., Cheng Y., Wang L., Ma D., Jing X., Wang F. (2006). Highly Efficient Electroluminescence from Green-Light-Emitting Electrochemical Cells Based on CuI Complexes. Adv. Funct. Mater..

[39] Armaroli N., Accorsi G., Holler M., Moudam O., Nierengarten J.-F., Zhou Z., Wegh R.T., Welter R. (2006). Highly Luminescent CuI Complexes for Light-Emitting Electrochemical Cells. Adv. Mater..

[40] Zhang Q., Komino T., Huang S., Matsunami S., Goushi K., Adachi C. (2012). Triplet Exciton Confinement in Green Organic Light-Emitting Diodes Containing Luminescent Charge-Transfer Cu(I) Complexes. Adv. Funct. Mater..

[41] Hernandez-Perez A.C., Vlassova A., Collins S.K. (2012). Toward a Visible Light Mediated Photocyclization: Cu-Based Sensitizers for the Synthesis of [5]Helicene. Org. Lett..

[42] Pirtsch M., Paria S., Matsuno T., Isobe H., Reiser O. (2012). [Cu(dap)_2_Cl] As an Efficient Visible-Light-Driven Photoredox Catalyst in Carbon–Carbon Bond-Forming Reactions. Chem. A Eur. J..

[43] Gong T., Adzima B.J., Baker N.H., Bowman C.N. (2013). Photopolymerization Reactions Using the Photoinitiated Copper (I)-Catalyzed Azide-Alkyne Cycloaddition (CuAAC) Reaction. Adv. Mater..

[44] Alzahrani A.A., Erbse A.H., Bowman C.N. (2013). Evaluation and development of novel photoinitiator complexes for photoinitiating the copper-catalyzed azide–alkyne cycloaddition reaction. Polym. Chem..

[45] Rehm D., Weller A. (1970). Kinetics of Fluorescence Quenching by Electron and H-Atom Transfer. Isr. J. Chem..

[46] Zhao J., Lalevée J., Lu H., MacQueen R., Kable S.H., Schmidt T.W., Stenzel M.H., Xiao P. (2015). A new role of curcumin: As a multicolor photoinitiator for polymer fabrication under household UV to red LED bulbs. Polym. Chem..

[47] Deng J., Chen W., Deng H. (2016). Synthesis of Dipyridyl Ketone Isonicotinoyl Hydrazone Copper(II) Complex: Structure, Anticancer Activity and Anticancer Mechanism. J. Fluoresc..

[48] Chew S.T., Lo K.M., Lee S.K., Heng M.P., Teoh W.Y., Sim K.S., Tan K.W. (2014). Copper complexes with phosphonium containing hydrazone ligand: Topoisomerase inhibition and cytotoxicity study. Eur. J. Med. Chem..

[49] Vafazadeh R., Moghadas Z., Willis A.C. (2015). Anion and solvent effects on the coordination behavior of N-(2-pyridinylmethylene)benzoylhydrazone with copper(II): Synthesis and structural characterization. J. Coord. Chem..

[50] Wong K.M.-C., Hung L.-L., Lam W.H., Zhu N., Yam V.W.-W. (2007). A Class of Luminescent Cyclometalated Alkynylgold (III) Complexes: Synthesis, Characterization, and Electrochemical, Photophysical, and Computational Studies of [Au (C∧ N∧ C)(C⋮CR)](C∧ N∧ C= κ3C, N, C Bis-cyclometalated 2, 6-Diphenylpyridyl). J. Am. Chem. Soc..

[51] Rajam B.M., Ramasamy P., Mahalingam U. (2017). Monodispersed gold nanoparticles as a probe for the detection of Hg2+ ions in water. Acta Chim. Slov..

[52] Ghosh D., Chattopadhyay N. (2013). Gold nanoparticles: Acceptors for efficient energy transfer from the photoexcited fluorophores. Opt. Photonics J..

[53] Mutlu S., Metin E., Yuksel S.A., Bayrak U., Nuhoglu C., Arsu N. (2020). In-situ photochemical synthesis and dielectric properties of nanocomposite thin films containing Au, Ag and MnO nanoparticles. Eur. Polym. J..

[54] Alsawafta M., Badilescu S., Paneri A., Truong V.-V., Packirisamy M. (2011). Gold-Poly(methyl methacrylate) Nanocomposite Films for Plasmonic Biosensing Applications. Polymers.

[55] Sun K., Pigot C., Chen H., Nechab M., Gigmes D., Morlet-Savary F., Graff B., Liu S., Xiao P., Dumur F. (2020). Free Radical Photopolymerization and 3D Printing Using Newly Developed Dyes: Indane-1,3-Dione and 1*H*-Cyclopentanaphthalene-1,3-Dione Derivatives as Photoinitiators in Three-Component Systems. Catalysts.

[56] Balaji D., Basavaraja S., Deshpande R., Mahesh D.B., Prabhakar B., Venkataraman A. (2009). Extracellular biosynthesis of functionalized silver nanoparticles by strains of Cladosporium cladosporioides fungus. Colloids Surf. B Biointerfaces.

[57] Burda C., Chen X., Narayanan R., El-Sayed M.A. (2005). Chemistry and Properties of Nanocrystals of Different Shapes. Chem. Rev..

[58] Shenashen M.A., El-Safty S.A., Elshehy E.A. (2014). Synthesis, Morphological Control, and Properties of Silver Nanoparticles in Potential Applications. Part. Part. Syst. Charact..

[59] Malval J.-P., Jin M., Balan L., Schneider R., Versace D.-L., Chaumeil H., Defoin A., Soppera O. (2010). Photoinduced Size-Controlled Generation of Silver Nanoparticles Coated with Carboxylate-Derivatized Thioxanthones. J. Phys. Chem. C.

[60] Zaier M., Vidal L., Hajjar-Garreau S., Balan L. (2017). Generating highly reflective and conductive metal layers through a light-assisted synthesis and assembling of silver nanoparticles in a polymer matrix. Sci. Rep..

[61] Aslan K., Lakowicz J.R., Geddes C.D. (2004). Tunable plasmonic glucose sensing based on the dissociation of Con A-aggregated dextran-coated gold colloids. Anal. Chim. Acta.

[62] Ghosh S.K., Pal T. (2007). Interparticle Coupling Effect on the Surface Plasmon Resonance of Gold Nanoparticles: From Theory to Applications. Chem. Rev..

[63] Nalawade P., Mukherjee P., Kapoor S. (2014). Triethylamine induced synthesis of silver and bimetallic (Ag/Au) nanoparticles in glycerol and their antibacterial study. J. Nanostructure Chem..

[64] Henglein A. (1989). Small-particle research: Physicochemical properties of extremely small colloidal metal and semiconductor particles. Chem. Rev..

[65] Belloni J. (1998). Contribution of radiation chemistry to the study of metal clusters. Radiat. Res..

[66] Kapoor S. (1998). Preparation, Characterization, and Surface Modification of Silver Particles. Langmuir.

[67] Ahmad T., Wani I.A., Manzoor N., Ahmed J., Asiri A.M. (2013). Biosynthesis, structural characterization and antimicrobial activity of gold and silver nanoparticles. Colloids Surf. B Biointerfaces.

[68] Bhuvanasree S., Harini D., Rajaram A., Rajaram R. (2013). Rapid synthesis of gold nanoparticles with Cissus quadrangularis extract using microwave irradiation. Spectrochim. Acta Part A Mol. Biomol. Spectrosc..

[69] Sundararajan B., Kumari B.R. (2017). Novel synthesis of gold nanoparticles using Artemisia vulgaris L. leaf extract and their efficacy of larvicidal activity against dengue fever vector *Aedes aegypti* L.. J. Trace Elem. Med. Biol..

[70] Zhi B., Song Q., Mao Y. (2018). Vapor deposition of polyionic nanocoatings for reduction of microglia adhesion. RSC Adv..

[71] Baganizi D.R., Nyairo E., Duncan S.A., Singh S.R., Dennis V.A. (2017). Interleukin-10 Conjugation to Carboxylated PVP-Coated Silver Nanoparticles for Improved Stability and Therapeutic Efficacy. Nanomaterials.

[72] Anyaogu K.C., Cai X., Neckers D.C. (2008). Gold nanoparticle photosensitized radical photopolymerization. Photochem. Photobiol. Sci..

[73] Kaur N., Singh J., Raj P., Singh N., Singh H., Sharma S.K., Kim D.Y., Kaur N. (2016). ZnO decorated with organic nanoparticles based sensor for the ratiometric selective determination of mercury ions. New J. Chem..

[74] Eisenhauer E.A., ten Bokkel Huinink W.W., Swenerton K.D., Gianni L., Myles J., Van der Burg M.E., Vermoken J.B., Bruser KColombo N. (1994). European-Canadian randomized trial of paclitaxel in relapsed ovarian cancer: High-dose versus low-dose and long versus short infusion. J. Clin. Oncol..

[75] Mohan S., Sundaraganesan N., Mink J. (1991). FTIR and Raman studies on benzimidazole. Spectrochim. Acta Part A Mol. Spectrosc..

[76] Sundaraganesan N., Ilakiamani S., Subramani P., Joshua B.D. (2007). Comparison of experimental and ab initio HF and DFT vibrational spectra of benzimidazole. Spectrochim. Acta Part A Mol. Biomol. Spectrosc..

[77] Baranwal B.P., Talat F., Varma A. (2009). Synthesis, spectral and thermal characterization of nano-sized, oxo-centered, trinuclear carboxylate-bridged chromium (III) complexes of hydroxycarboxylic acids. J. Mol. Struct..

[78] Kolmas J., Jaklewicz A., Zima A., Buæko M., Paszkiewicz Z., Lis J., OElósarczyk A., Kolodziejski W. (2011). Incorporation of carbonate and magnesium ions into synthetic hydroxyapatite: The effect on physicochemical properties. J. Mol. Struct..

