# Supplementary material for: Copper II Complexes Based on Benzimidazole Ligands as a Novel Photoredox Catalysis for Free Radical Polymerization Embedded Gold and Silver Nanoparticles
Source: Polymers (Basel). 2023 Mar 3;15(5):1289. doi: 10.3390/polym15051289 (PMC10007263; doi:10.3390/polym15051289)
Supplement: Supplementary file 1 [file polymers-15-01289-s001.zip › polymers-2189470-supplementary.pdf]

polymers

MDPI

Copper II Complexes Based on Benzimidazole Ligands as a Novel Photoredox Catalysis for Free Radical Polymerization Embedded Gold and Silver Nanoparticles

Lama M. Alhomaidan [1], Haja Tar [1,*], Abrar S. Alnafisah [1,*], Lotfi M. Aroua [1,2], Noura KouKi [1], Fahad M. Alminderej [1] and Jacques Lalevee [3]

1 Department of Chemistry, College of Science, Qassim University, Buraidah 51452, Saudi Arabia
2 Laboratory of Organic Structural Chemistry and Macromolecules, Department of Chemistry, Faculty of Sciences of Tunis, Tunis El-Manar University, El Manar I, Tunis 2092, Tunisia
3 CNRS, IS2M UMR 7361, Université de Haute-Alsace, F-68100 Mulhouse, France
* Correspondence: h.tar@qu.edu.sa (H.T.); alnafisaha@qu.edu.sa (A.S.A.)

Synthesis

The synthesis of ligands ((benzimidazol-2-phenyl)iminomethyl)phenol **(HL1) (1)** and 2-(1-*H*-benzimidazol-2-yl)phenyl)imino)methyl) naphthol **(HL2) (2)** was achieved via the condensation of 2-(1*H*-Benzimizadol-2-yl)aniline with salicylaldehyde or naphthylaldehyde according to the literature conditions [73,74] following the Scheme 1.

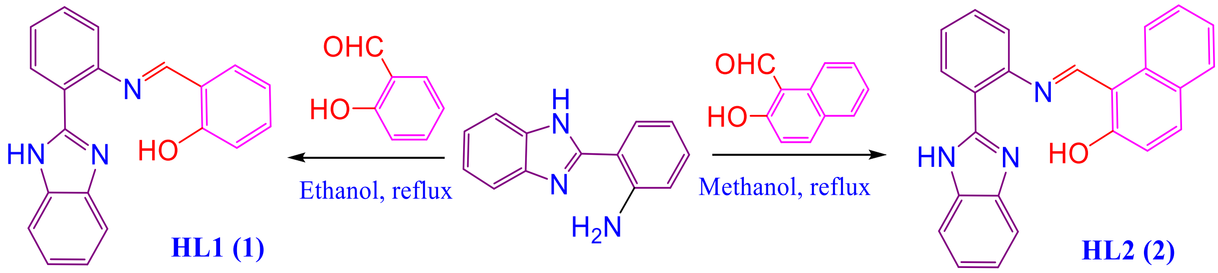

Scheme S1: Synthesis of Ligands **HL1 (1)** and **HL2 (2)**

Mixed complexes **(3)** was synthesized by reacting in equimolar molar amount of ethanolic solution **HL1 (1)**, ethanolic solution of copper salt ($CuCl_2.2H_2O$) and 2-mercaptobenzimidazole (Scheme 2**)**. The same protocol was adopted to the synthesis of complexes **(4)** from 2-(1-*H*-benzimidazol-2-yl)phenyl)imino)methyl) naphthol **HL2 (2)** (Scheme 2). All new obtained complexes were characterized by spectroscopic data of FT-IR spectroscopy, UV-Visible electronic absorption, thermal analysis and X-ray powder diffraction. The analytical data was resumed in Table 1.

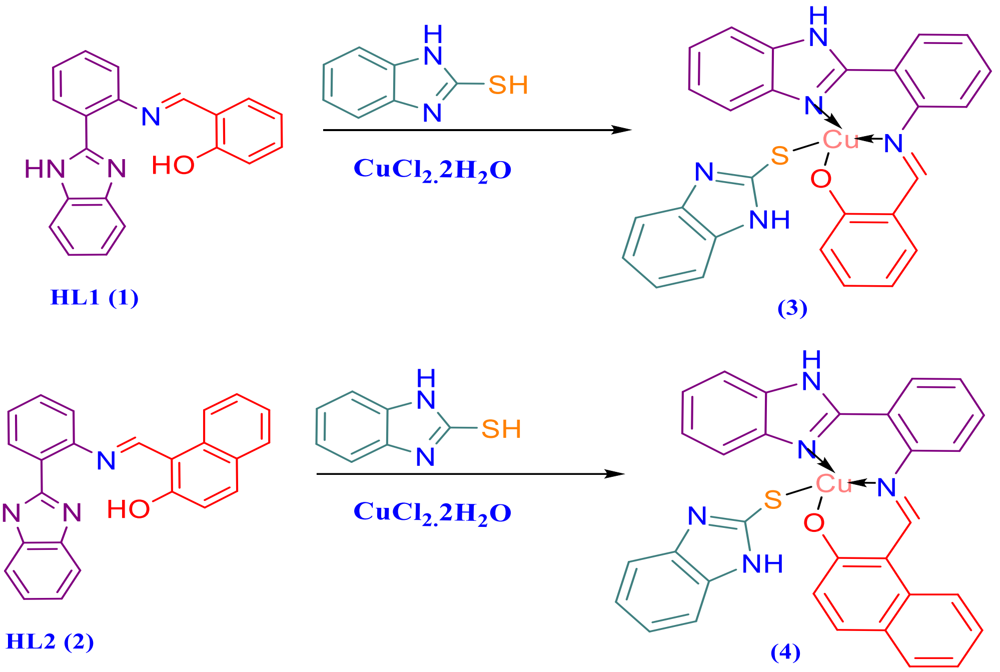

Scheme S2: Structure of mixed complexes **(3)** and **(4)**

Table S1: Analytical data of mixed synthesized complexes

Entry	Complex	Colour	Empirical formula	Yield (%)	Mp(°C)
1	**3**	Green Pistache	$C_{27}H_{19}CuN_5OS$	82	183-185
2	**4**	Light Green pistache	$C_{31}H_{21}CuN_5OS$	85	215-217

Infrared spectra

The IR data of free ligands and their copper complexes were reviewed in Table 2. The ligands **HL1 (1)** and **HL2 (2)** exhibited the existence of broad bands at 3398 and 3385 cm^{-1} respectively assigned to the phenolic hydroxyl group vibration. In the case of complexes (**3**) and (**4**), the spectra indicate the absence of this vibration which corresponds to the deprotonation of the hydroxyl group in the formation processes of complexes. The spectra of **HL1 (1)** manifest also another vibration at 3252 cm^{-1} attributed to the υ(NH) group of benzimidazole group. This band was shifted at a lower frequency in the complex **(3)** and **(4) and appeared respectively at 3134 and 3145 cm^{-1}.** The spectra of **HL1 (1)** displayed eventually the presence of band at 1664 **cm^{-1}** related to azomethine moiety. This band deviate in the complexes **(3)** and **(4)** and shifted to lower frequencies (1623, 1606 cm^{-1}) that is in a good agreement with the previously reported data [75, 76]. This mode of chelation is supported by the appearance of new bands in the (496, 498) and (561, 562) cm^{-1} attributed to ν(O→M) and ν(N→M) respectively (Table 2). These bands were absent in the spectra of the ligand, thus confirming the participation of oxygen, nitrogen and sulfur atoms in the coordination.

Table S2: The vibrational assignment wavenumbers in cm^{-1} of ligands and mixed complexes

complex	ν(OH)	ν(N-H)$_{benzim}$	ν(C=N)	ν(M-O)	ν(M-N)
**HL1 (1)**	3398	3252	1664	-	-
**HL2 (2)**	3385	3235	1634	-	-
**Complex (3)**	-	3134	1623	496	562
**Complex (4)**	-	3145	1606	498	595

Thermal analysis study

The study of thermal decomposition of copper complexes was achieved at room temperature. Two endothermic decomposition stages principally observed in the thermograph degradation of mixed complex **(3)**. The first endothermic stage at 241-332°C is responsible for the desorption of mercaptobenzimidazole with mass loss amounting to 27.88%. In the range of 475-577 °C, the second endothermic step is assigned to the elimination phenol with a mass loss amounting to 16.25 %. The thermal decomposition of complex **(4)** present essentially an endothermic stage at 310-414 °C with a mass loss 20.83% corresponding to the detachment of mercaptobenzimidazole.

X-ray powder diffraction study

X-ray powder diffraction study of metal hybrid complexes was provided to determine the type of crystal system, lattice parameters, 2θ range and crystallinity size, and the cell volume. From the indexed data, the unit cell parameters were also calculated and are listed in Table 3. The powder XRD patterns of synthesized compounds possess triclinic structure for complexes (3), while complex (4) has monoclinic structure. The crystal structures of similar type of samples were reported as monoclinic and orthorhombic [77, 78].

Table S3: X-ray powder diffraction crystal data of complexes: Lattice constant, inter axial angle, Crystal system, unit cell volume, 2θ range and cristallinity size of different metal complex

Parameters		Complex (3)	Complex (4)
**Empirical formula**		$C_{27}H_{19}CuN_5OS$	$C_{31}H_{21}CuN_5OS$
**Lattice constant**	a (Å)	8.926	16.696
	b (Å)	6.425	5.216
	**c (Å)**	16.99	9.529
**Inter axial angle**	**α (°)**	85.63	90.00
	**β (°)**	100.91	98.42
	**γ (°)**	99.81	90.00
**Crystal system**		a≠b≠c, α≠ β≠ γ ≠90	a≠b≠c, α= γ =90, β≠90
		Triclinic	Monoclinic
**Unit cell Volume (Å^3)**		942	820.9
**2θ range**		5.26-60.60	5.28-39.28
**Crystallite Size**		462	410
**Density (gcm^{-3})**		1.590	1.573

Experimental

FT-IR spectra were achieved using Agilent FT-IR Spectrometer Cary 600 with microscope in the range of 400-4000 cm^{-1}. The ^{1}H and ^{13}C NMR spectra were recorded on a 400 MHz NMR spectrometer Bruker Avance at 400 and 100 MHz, respectively. All spectra were obtained using DMSO *d6* as solvent and referenced to TMS. Chemical shifts of ^{1}H NMR spectra are reported in parts per million (ppm) on the *δ* scale from an internal standard of residual DMSO (2.50 ppm). Data are reported as follows: chemical shift, integration, multiplicity (s =singlet, d = doublet, t = triplet, q = quartet, and m = multiplet) and coupling constant in hertz (Hz). Chemical shifts of ^{13}C NMR spectra are reported in ppm from the central peak of DMSO d_6 (39.52 ppm) on the *δ* scale. The

elemental analysis was carried out on Flash *Smart* Elemental Analyzer Thermo Fisher Scientific performing carbon, hydrogen, nitrogen analyses.

The melting points were measured in open capillary tubes using The Stuart SMP30 Apparatus. Analytical TLC was performed using Silica Gel 60 F254 plates (Sigma 40–60 µm). The developed chromatogram was visualized under UV lamp (254 nm). All commercially available reagents were purchased from Sigma-Aldrich and used without further purification. Absolute ethanol was used as solvent. All reactions were carried using oven-dried glassware unless otherwise stated.

Preparation of ((benzimidazol-2-phenyl)iminomethyl)phenol (HL1) (1)

A solution of 2-(1*H*-benzoimidazol-2-yl)aniline (1.05 g, 5mmol) in 35 mL of methanol was heated gradually at 65 °C for 30min. Then, a solution of salicylaldehyde (0.67 g, 5.5 mmol) dissolved in 10 mL of methanol was added and the mixture was refluxed for 4h. After the end of the reaction, the mixture was cooled and the solvent was removed under reduced pressure. The residue was purified by recrystallization in ethanol to give as a pale yellow crystal powder. Described data agreed with that reported [73].

Yield: 78%., ^{1}H NMR (400MHz, DMSO-*d6*), *δ*(ppm): 10.18 (s,1H), 7.94 (s,1H), 7.64 (d, 1H), 7.15 (m, 7H), 6.89 (dd, 2H), 6.80 (t, 1H) , 6.65 (m, 2H). ^{13}C NMR (100 MHz, DMSO-d*6*) *δ*(ppm): 154.77, 147.81, 144.35, 143.97, 133.36, 131.98, 130.46, 126.95, 126.57, 125.05, 122.60, 122.43, 119.73, 119.06, 118.29, 116.29, 115.24, 112.03, 110.68, 63.50. IR (KBr pellets, cm^{-1}): 3398.67 (O-H). 3252.22 (N-H), 1651 (C=C), 1664 (C=N), 1298.83 (C-N). Elemental analysis For $C_{20}H_{16}N_{3}O$ calcd.: C, 76.58; H, 4.98; N, 13.32%. Found: C, 76.66; H, 4.82; N, 13.41%. HRMS for $C_{20}H_{16}N_{3}O$ calcd.: 314.1288, Found: 314.1289. Described data agreed with that reported.

Preparation of 2-(1-H-benzimidazol-2-yl)phenyl)imino)methyl) naphthol (HL2) (2)

To a solution of 2-(1H-benzoimidazol-2-yl)aniline (1.05 g, 5 mmol) dissolved in 50 mL of absolute ethanol, was added a solution of 2-hydroxy-1-naphthaldehyde (0.86 g, 5 mmol) dissolved in 20 mL of ethanol and three drop of acetic acid were added. Then, the mixture left at reflux for 5 h. A t the end of the reaction, the volume was reduced. Diethyl ether was added and the solution was left in refrigerator. Light yellow crystals collected, filtered, washed with diethyl ether and dried. Described data agreed with that reported [74].

Yield: 75%. IR (KBr pellets, cm^{-1}): *ν*C=N: 1634. ^{1}H NMR ($CDCl_3$, 400 MHz), *δ* (ppm): 14.43 (s, 1H, OH), 9.41 (s, 1H, CH=N), 7.95 (d, 1H, Ar-H), 7.88 (s, 1H, Ar-H), 7.78 (d, 1H, Ar-H), 7.68 (m, 1H, Ar-H), 7.53 (s, 1H, Ar-H), 7.50 (s, 1H, Ar-H), 7.45 (s, 1H, Ar-H), 7.40 (s, 1H, Ar-H), 7.34 (m, 1H, Ar-H), 7.01 (d, 1H, Ar-H). ^{13}C NMR ($CDCl_3$, 75. MHz), *δ* (ppm): 166.31, 158.85, 136.14, 132.35, 131.47, 129.23, 128.08, 127.57, 126.66, 123.65, 123.01, 120.54, 119.84, 119.54, 119.17, 109.75. Elemental analysis for $C_{25}H_{19}N_{3}0$, Calcd.: C, 79.56; H, 5.08; N, 11.11%. Found: C, 79.78; H, 4.99; N, 10.99%.

Preparation of mixed complex (3)

To a solution of **HL1 (1)** (0.43 g, 1.377 mmol) in 10 mL of absolute ethanol was added ethanolic solution of copper chloride dehydrate (0.234g, 1.377 mmol). The reaction mixture was stirred at room temperature for 30 mn. A solution of 2-mercaptobenzimidazole **(**0.20g, 1.377 mmol) in 10mL of absolute ethanol was added. The reaction mixture was refluxed for 4 h. The progress of reaction was monitored with TLC (DMF/ethanol: 20/80). At the end of the reaction, the mixture was cooled, filtered, washed with ethanol and then with small portion of DMF.

Green pistache Yield: 82%, Mp: 183-185°C, UV-Vis (λmax):; IR (υ, Cm^{-1}) : 3134 (m), 2970 (m), 1623 (m), 1588 (s), 1567 (s), 1532(m), 1507 (s), 1362 (m), 1346 (s), 1274 (m), 1218 (m), 1183 (m), 1155 (m), 1088 (m), 1051 (s), 1012(m), 862 (m), 825 (m), 732 (m), 653 (m), 597 (m), 562 (m), 535 (m), 496 (m), 421 (m). Elemental analysis for C27H19CuN5OS calcd.: C, 61.76; H, 3.65; N, 13.34. Found: C, 61.73; H, 3.62; N, 13.33%. µeff 75 (BM): 1.73. Molar conductance ($\Omega^{-1}cm^{2}$ mol^{-1}): 14.5.

Preparation of mixed complex (4)

To ethanolic solution of **HL2 (2)** (0.5 g, 1.377 mmol) was added a solution of copper chloride dehydrate (0.234g, 1.377 mmol) in 10 mL of absolute ethanol. The reaction stirred for 30 mn at room temperature and a solution of 2-mercaptobenzimidazole **(**0.20g, 1.377 mmol) in 10mL of ethanol was added. After the complete of addition,

reaction mixture was refluxed for 4 h. The progress of reaction was controlled with TLC (DMF/ethanol: 20/80). At the end of the reaction, the mixture was cooled, filtered, washed with ethanol and then with small portion of DMF.

Light green pistache, Yield: 85%, Mp: 215-217°C, UV-Vis (λmax): ; IR (υ, cm^{-1}) : 3145 (m), 3032 (m), 1619 (m), 1504 (s), 1567 (s), 1455(m), 1402 (s), 1344 (m), 1258 (s), 1216 (m), 1180 (m), 1089 (m), 1050 (m), 1012 (m), 1050 (s), 1012(m), 859 (m), 731 (m), 650 (m), 617 (m), 595 (m), 498 (m), 472 (m), 413 (m). Elemental analysis for C31H21CuN5OS calcd.: C, 64.74; H, 3.68; N, 12.18. Found: C, 64.71; H, 3.66; N, 12.15%. μeff 75 (BM): 1.73. Molar conductance (Ω−1cm^{2} mol^{-1}): 13.8.

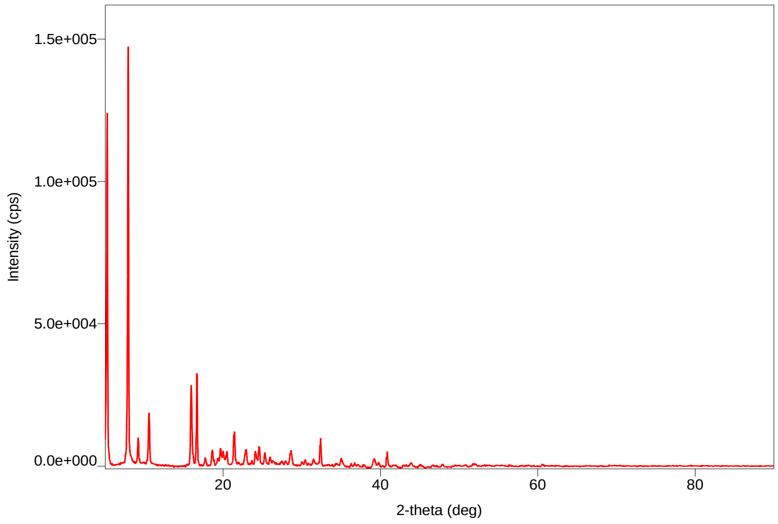

XRD pattern of complex 3

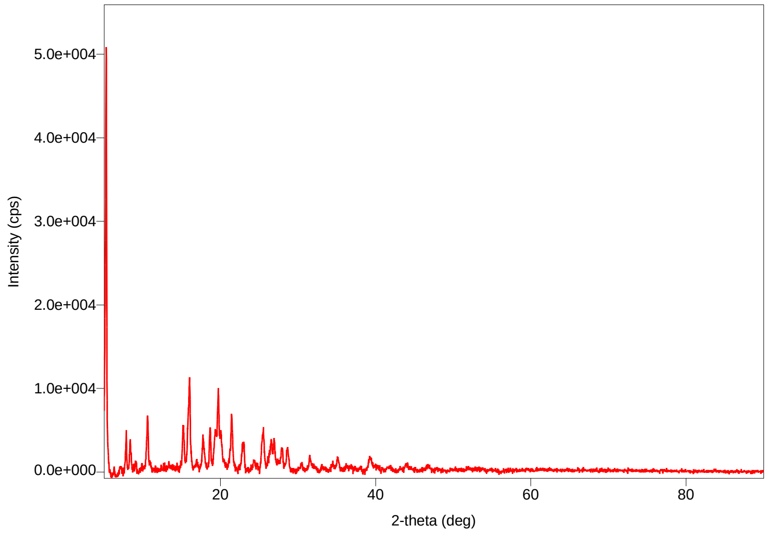

XRD pattern of complex 4

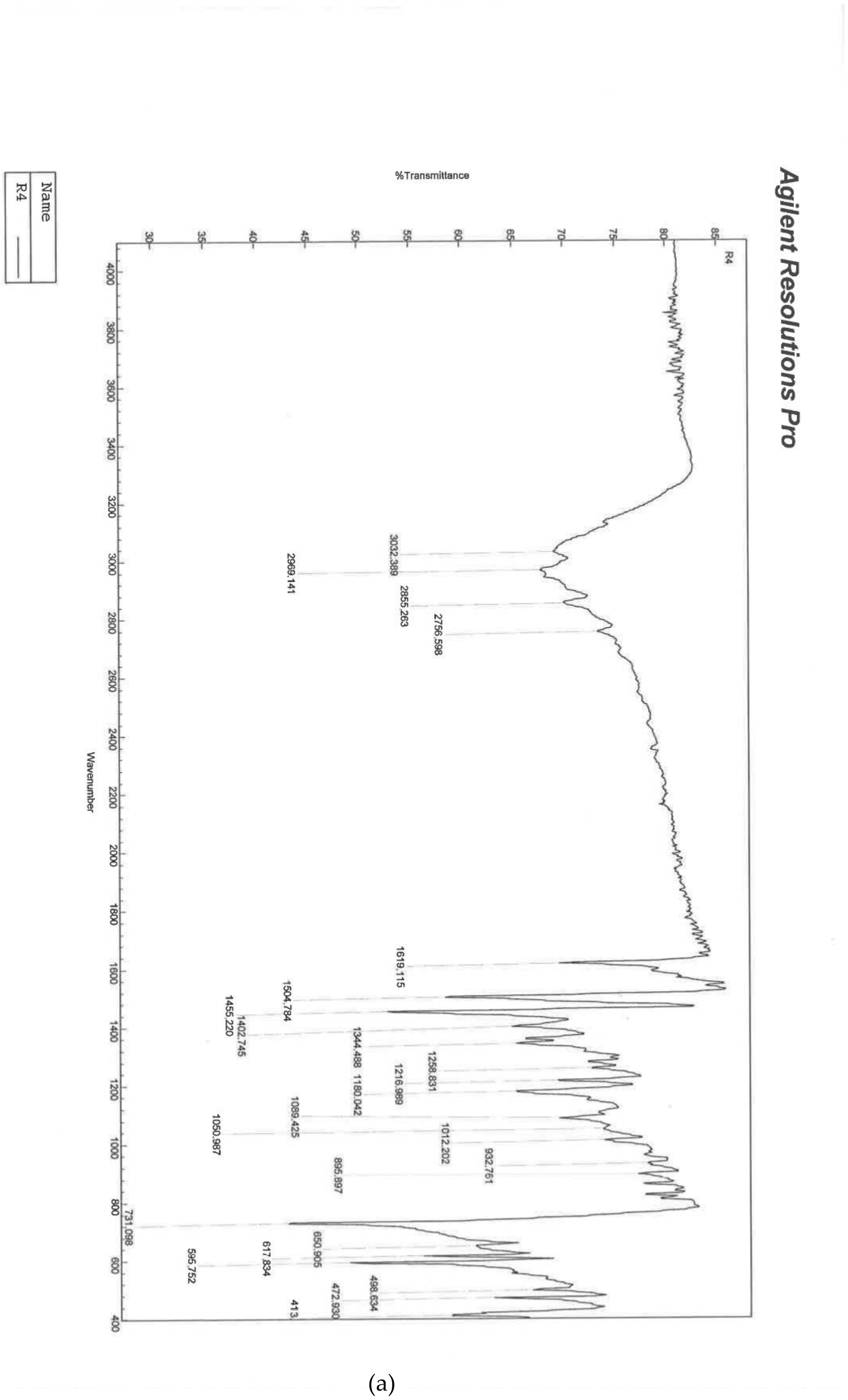
Agilent Resolutions Pro
%Transmittance
Wavenumber
R4
Name
R4
3032.389
2969.141
2855.263
2756.598
1619.115
1504.784
1455.220
1402.745
1344.488
1258.831
1216.989
1180.042
1089.425
1050.987
1012.202
932.761
895.897
731.098
650.905
617.834
595.752
498.634
472.930
413

(a)

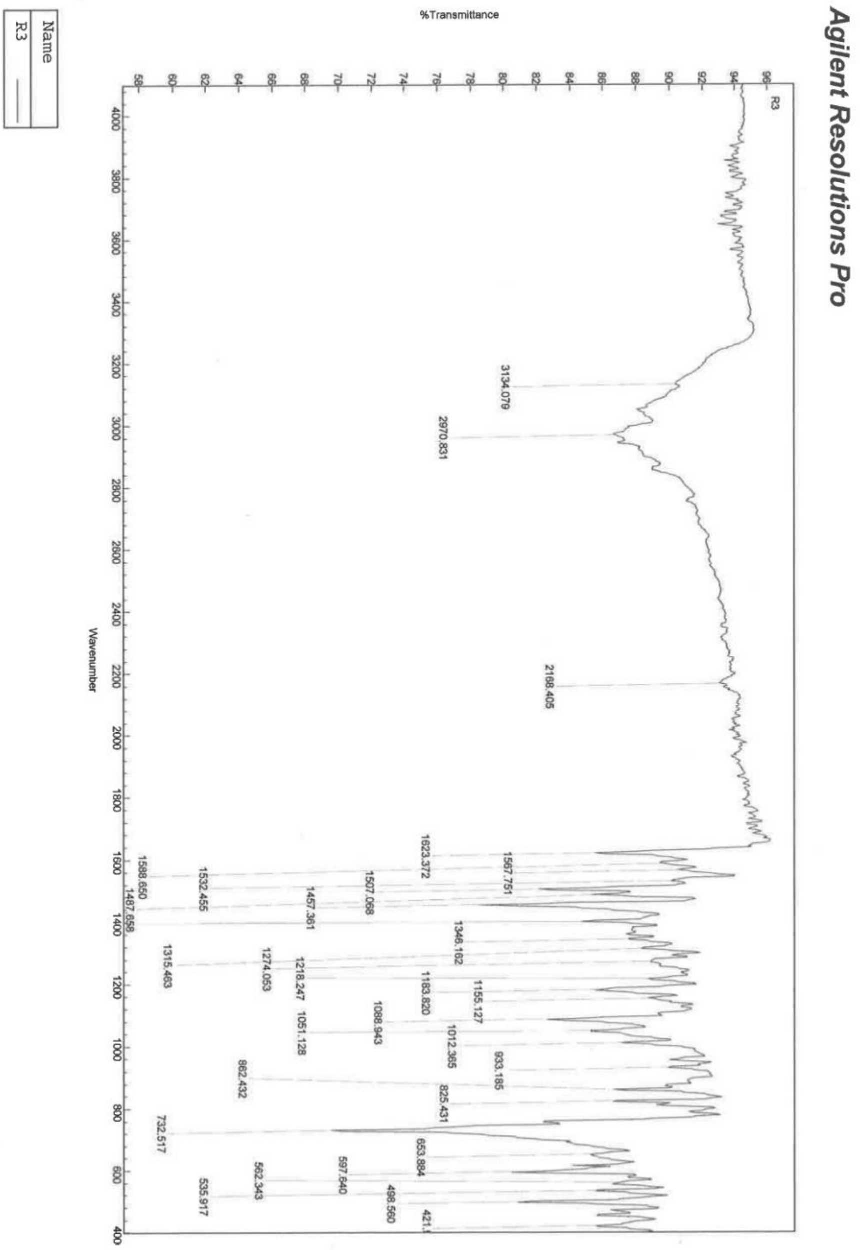

(b)

IR spectrum of the copper complexes

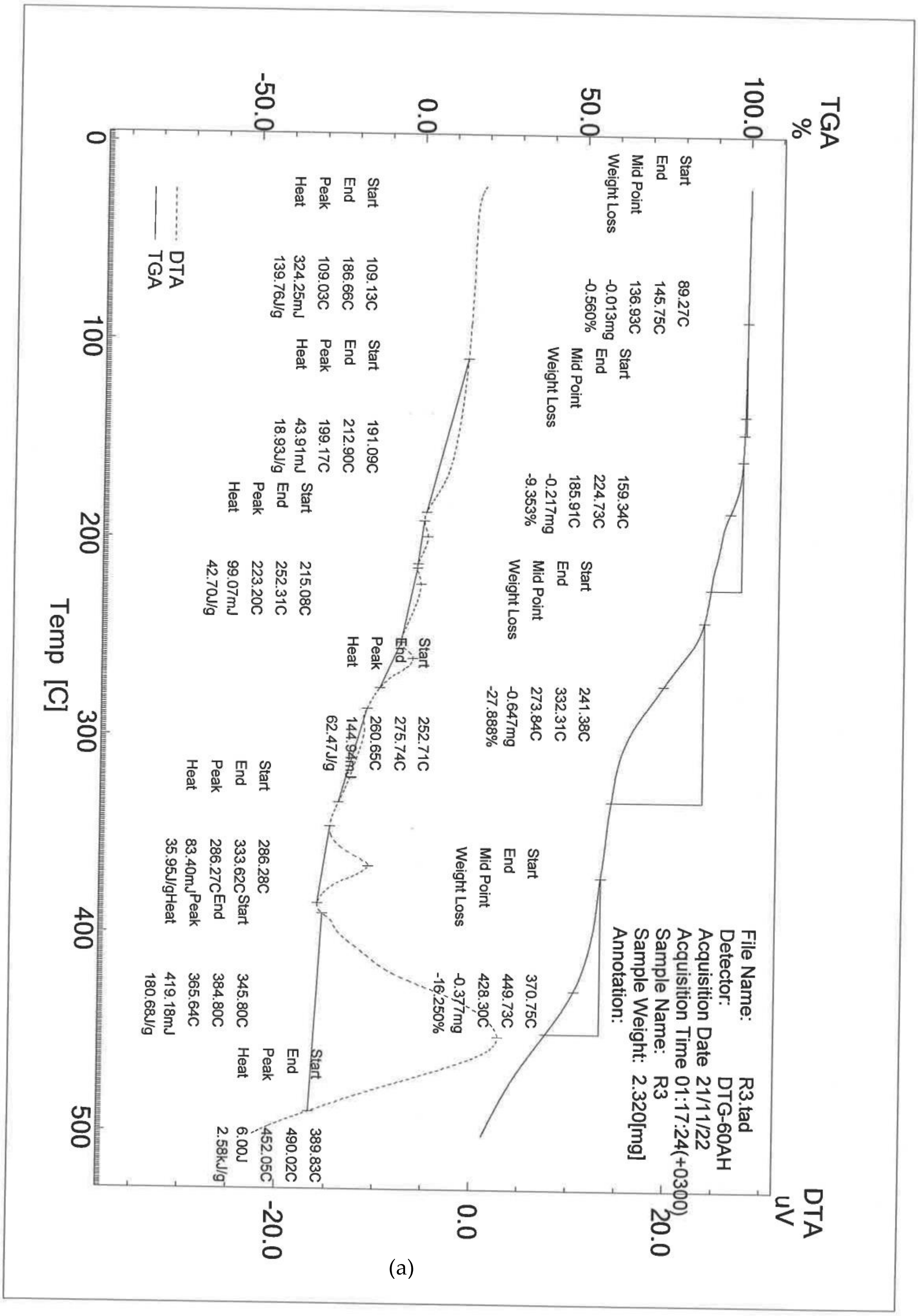
TGA
%
DTA
uV
Temp [C]
File Name: R3.tad
Detector: DTG-60AH
Acquisition Date 21/11/22
Acquisition Time 01:17:24(+0300)
Sample Name: R3
Sample Weight: 2.320[mg]
Annotation:
DTA
TGA

(a)

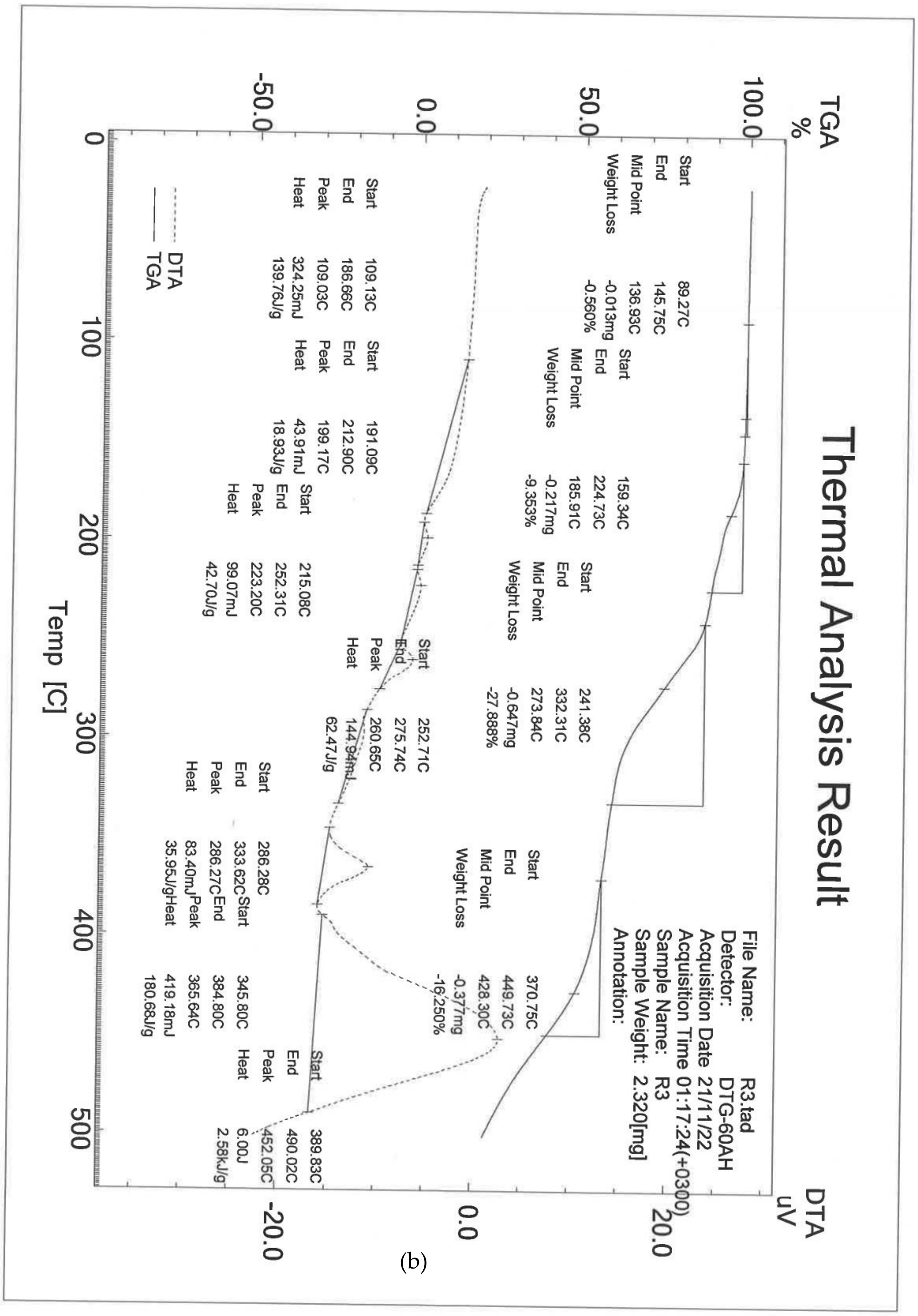

(b)

DTA, TGA and DrTGA of the complexes.

Copper Complex Oxidation Process

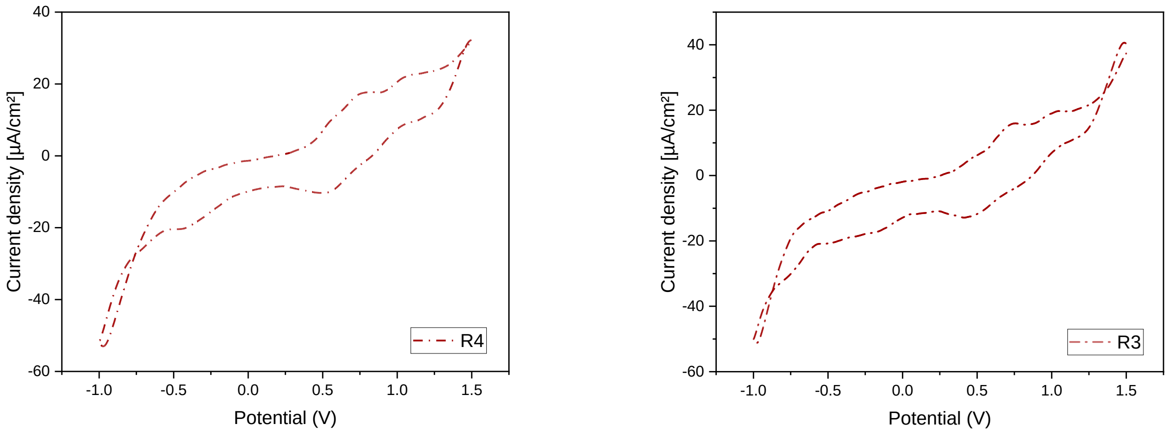

(**a**) (**b**)

Figure S1. Cyclic voltammogram of Cu complexes in Acetonitrile (a) R4 complex and (b) R3 complex.

2.7. Fluorescence Experiments

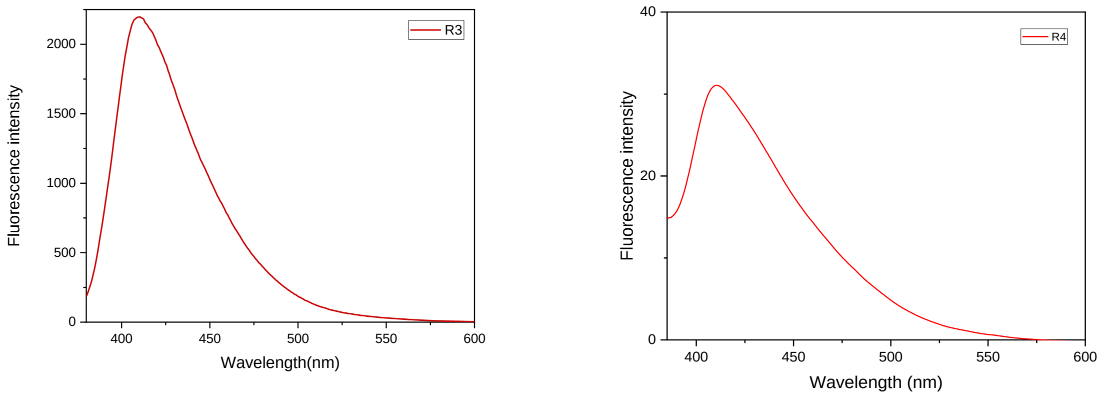

(**a**) (**b**)

Figure S2. Luminescence spectra of R3 (a), and R4 (b) complexes in DMF concentration of 10^{-4} M.

3.2. Photoinduced Synthesis of Au Nanoparticles

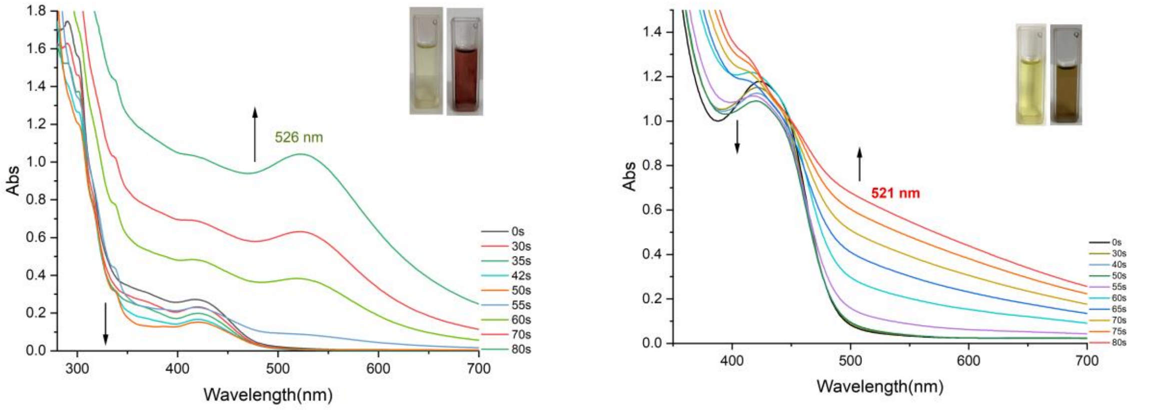

(**a**) (**b**)

Figure S3. Evolution of the absorption spectra of the irradiated mixtures (λirr = 405 nm). Solution: (R3 (a) and R4 (b)) 1X10⁻⁴ M gold chloride 3wt% and TEA 0.2 wt% dissolved in 25 mL of DMF.

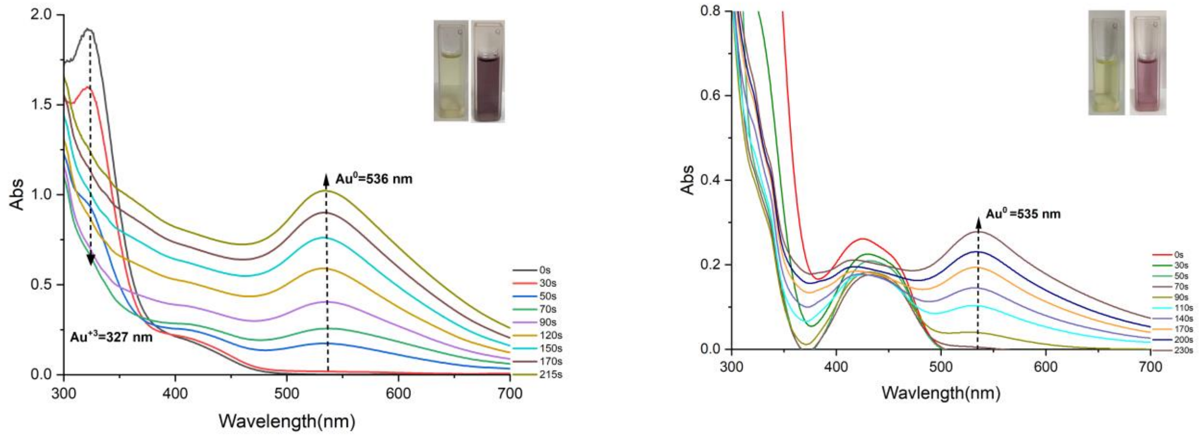

(**a**) (**b**)

Figure S4. Evolution of the absorption spectra of the irradiated mixtures (λirr = 405 nm). Solution: (R3 (a) and R4 (b)) 1X10⁻⁴ M gold chloride 3wt% and iodonium salt 1 wt% dissolved in 25 mL of DMF.

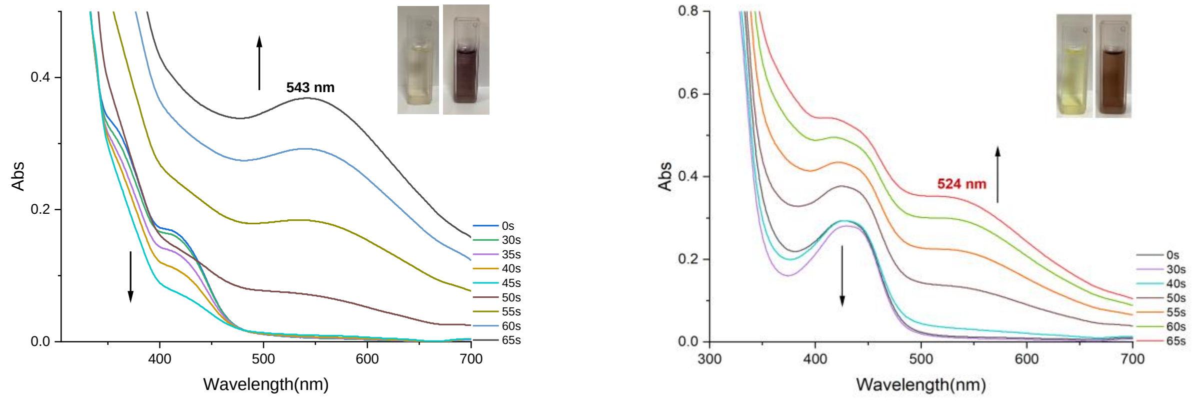

(**a**) (**b**)

Figure S5: Evolution of the absorption spectra of the irradiated mixtures (λirr = 405 nm). Solution: (R3 (a) and R4 (b)) 1X10^{-4} M gold chloride 3wt%, TEA 0.2wt%, iodonium salt 1 wt% dissolved in 25 mL of DMF.

3.3. *Photoinduced Synthesis of Ag Nanoparticles*

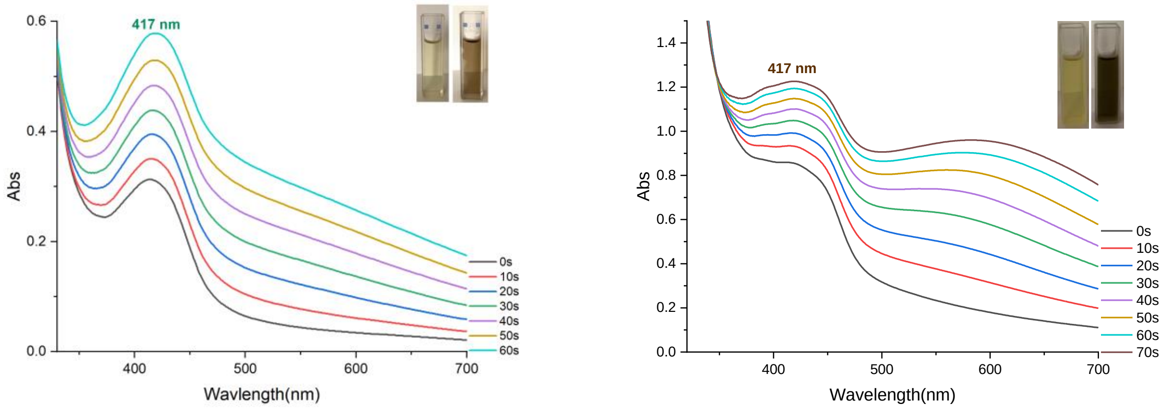

(**a**) (**b**)

Figure S6: Evolution of the absorption spectra of the irradiated mixtures (λ_{irr} = 405 nm). Solution (R3 (a) and R4 (b)) 1X10^{-4} M $AgNO_3$ 2wt% and TEA 0.2 wt% dissolved in 25 mL of DMF.

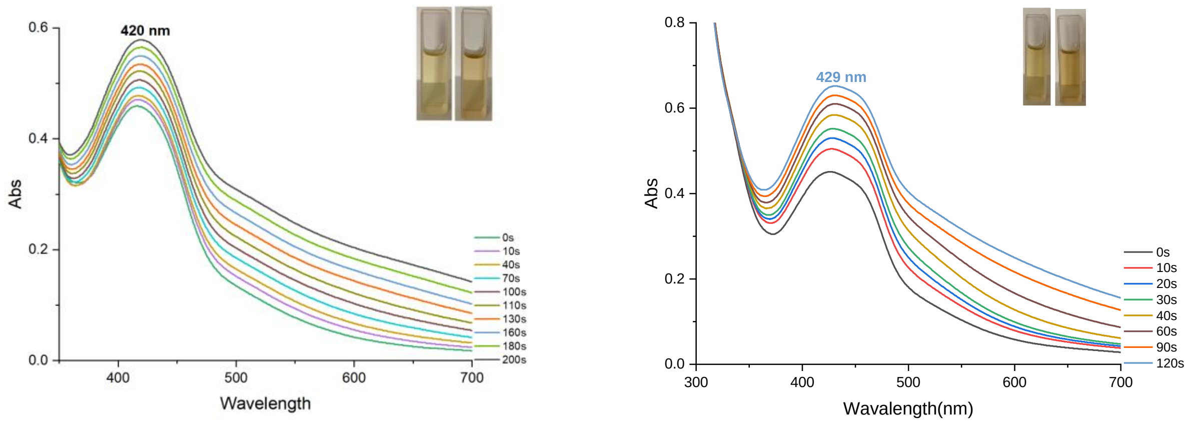

(**a**) (**b**)

Figure S7: Evolution of the absorption spectra of the irradiated mixtures (λ_{irr} = 405 nm). Solution (R3 (a) and R4 (b)) 1X10^{-4} M $AgNO_3$ 2wt% iodonium salt 1 wt% dissolved in 25 mL of DMF.

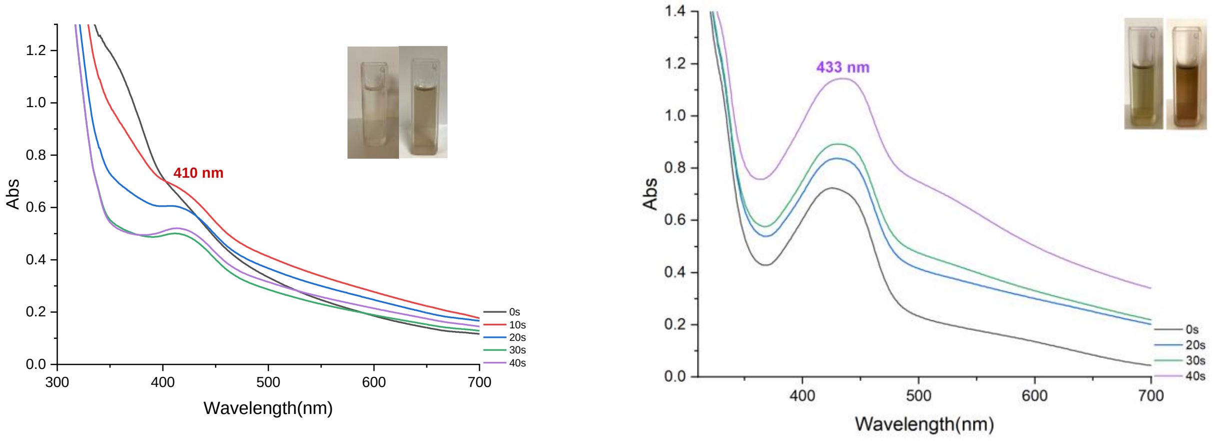

(**a**) (**b**)

Figure S8: Evolution of the absorption spectra of the irradiated mixtures (λ_{irr} = 405 nm). Solution (R3 (a) and R4 (b)) 1X10^{-4} M $AgNO_3$ 2wt%, iodonium salt 1 wt%, and TEA 0.2 wt% dissolved in 25 mL of DMF.

3.4. Fabrication of AuNPs and AgNPs Embedded Polymer

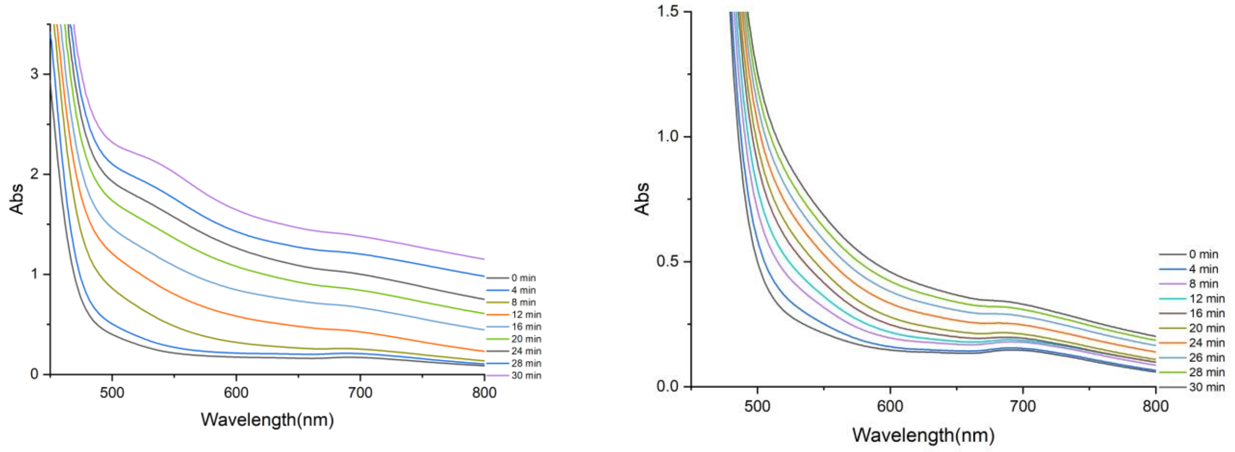

(**a**) (**b**)

Figure S9. Time evolution of the absorption spectrum of the ethylene glycol diacrylate (EGDA) during photopolymerization, irradiated mixtures (λ_{irr} = 405 nm). Solution: R3 in (a)and R4 in (b) 1X10^{-4} M gold chloride 3wt%, TEA 0.2wt%, and iodonium salt 1 wt% dissolved in 95.6wt%.

References

73 Kaur, N., Singh, J., Raj, P., Singh, N., Singh, H., Sharma, S. K., Kim D. Y., Kaur, N. ZnO decorated with organic nanoparticles based sensor for the ratiometric selective determination of mercury ions. New J. Chem., 40(2), 1529-1534

74 Eisenhauer, E. A., ten Bokkel Huinink, W. W., Swenerton, K. D., Gianni, L., Myles, J., Van der Burg, M. E., Vermoken J. B., Bruser K. Colombo, N. European-Canadian randomized trial of paclitaxel in relapsed ovarian cancer: high-dose versus low-dose and long versus short infusion. J. Clinical Oncology, 12(12), 2654-2666(1994).

75 Mohan, S., Sundaraganesan, N., Mink, J. FTIR and Raman studies on benzimidazole. Spectrochimica Acta Part A: Molecular Spectroscopy, 47(8), 1111-1115 (1991).

76 Sundaraganesan, N., Ilakiamani, S., Subramani, P., Joshua, B. D. Comparison of experimental and ab initio HF and DFT vibrational spectra of benzimidazole. Spectrochimica Acta Part A: Molecular and Biom. Spectro., 67(3-4), 628- 635 (2007).

77 Baranwal, B. P., Talat, F., Varma, A., 2009. Synthesis, spectral and thermal characterization of nano-sized, oxo-centered, trinuclear carboxylate-bridged chromium (III) complexes of hydroxycarboxylic acids. J. Mol. Struct. 920, 472-477. doi:10.1016/j.molstruc.2008.12.029

78 Kolmas J., Jaklewicz A., Zima A., Buæko M., Paszkiewicz Z., Lis J., OElósarczyk A., Kolodziejski W., 2011. Incorporation of carbonate and magnesium ions into synthetic hydroxyapatite: the effect on physicochemical properties. J. Mol. Struct. 987, 40-50. doi: 10.1016/ j.molstruc. 2010.11.058
